# Discovery of a novel EGFR ligand DPBA that degrades EGFR and suppresses EGFR-positive NSCLC growth

**DOI:** 10.1038/s41392-020-00251-2

**Published:** 2020-10-09

**Authors:** Nan Yao, Chen-Ran Wang, Ming-Qun Liu, Ying-Jie Li, Wei-Min Chen, Zheng-Qiu Li, Qi Qi, Jin-Jian Lu, Chun-Lin Fan, Min-Feng Chen, Ming Qi, Xiao-Bo Li, Jian Hong, Dong-Mei Zhang, Wen-Cai Ye

**Affiliations:** 1 College of Pharmacy, Jinan University, Guangzhou, China; 2 Guangdong Province Key Laboratory of Pharmacodynamic Constituents of Traditional Chinese Medicine and New Drugs Research, Jinan University, Guangzhou, China; 3 School of Medicine, Jinan University, Guangzhou, China; 4 State Key Laboratory of Quality Research in Chinese Medicine, Institute of Chinese Medical Sciences, University of Macau, Macao, China

**Keywords:** Drug development, Lung cancer

## Abstract

Epidermal growth factor receptor (EGFR) activation plays a pivotal role in EGFR-driven non-small cell lung cancer (NSCLC) and is considered as a key target of molecular targeted therapy. EGFR tyrosine kinase inhibitors (TKIs) have been canonically used in NSCLC treatment. However, prevalent innate and acquired resistances and EGFR kinase-independent pro-survival properties limit the clinical efficacy of EGFR TKIs. Therefore, the discovery of novel EGFR degraders is a promising approach towards improving therapeutic efficacy and overcoming drug resistance. Here, we identified a 23-hydroxybetulinic acid derivative, namely DPBA, as a novel EGFR small-molecule ligand. It exerted potent in vitro and in vivo anticancer activity in both EGFR wild type and mutant NSCLC by degrading EGFR. Mechanistic studies disclosed that DPBA binds to the EGFR extracellular domain at sites differing from those of EGF and EGFR. DPBA did not induce EGFR dimerization, phosphorylation, and ubiquitination, but it significantly promoted EGFR degradation and repressed downstream survival pathways. Further analyses showed that DPBA induced clathrin-independent EGFR endocytosis mediated by flotillin-dependent lipid rafts and unaffected by EGFR TKIs. Activation of the early and late endosome markers rab5 and rab7 but not the recycling endosome marker rab11 was involved in DPBA-induced EGFR lysosomal degradation. The present study offers a new EGFR ligand for EGFR pharmacological degradation and proposes it as a potential treatment for EGFR-positive NSCLC, particularly NSCLC with innate or acquired EGFR TKI resistance. DPBA can also serve as a chemical probe in the studies on EGFR trafficking and degradation.

## Introduction

Lung cancer is the most common cancer and the leading cause of cancer-related death, and non-small cell lung cancer (NSCLC) accounts for 84% of all lung cancer diagnoses.^1^ Epidermal growth factor receptor (EGFR) is often overexpressed in NSCLC.^2^ EGFR tyrosine kinase inhibitors (TKIs) have been widely used to treat EGFR-positive NSCLC. Although EGFR TKIs have demonstrated remarkable clinical efficacy, they may also invariably induce acquired resistance. Most patients present with a T790M mutation after gefitinib treatment.^3^ The second-generation EGFR TKI afatinib irreversibly binds to EGFR, but it lacks selectivity for EGFR WT and EGFR T790M and provokes adverse reactions.^4^ The third-generation EGFR TKI osimertinib is effective in patients with the T790M mutation.^5^ However, certain patients develop other acquired resistances such as a C797S mutation.^6^ The inhibition of EGFR kinase activity may result in “kinome rewiring”, which, in turn, causes compensatory feedback activation of alternative kinases.^7^ Moreover, 10–20% of NSCLCs with EGFR mutation are insensitive to EGFR TKIs and most NSCLCs with EGFR WT do not respond to TKIs despite EGFR up-regulation. This response is a manifestation of innate resistance.^8,9^ Recent studies reveal that EGFR kinase-independent activity also promotes cancer cell survival and chemoresistance.^10–12^ Hence, targeting EGFR by inducing degradation rather than inhibition of kinase activity could be a more effective and complete approach to repress EGFR in NSCLC treatment.

Several EGFR-degrading strategies have been reported. One approach is to deliver specific EGFR small interfering RNAs (siRNAs) in vivo. However, their short half-life, rapid degradability, and off-target effects have reduced their relative efficacy.^13,14^ A more promising strategy is to target EGFR degradation with chemicals. Proteolysis-targeting chimera (PROTAC) technology has been used in EGFR degradation and has entailed the connexion of EGFR TKIs with E3 ligase ligands to form ternary chimeras for proteasomal degradation.^15,16^ However, the optimization of solubility, membrane permeability, and metabolic stability increase challenge to make these ternary chimeras druggable.^17^ EGFR degradation has been induced by several small molecules, although they do not directly target EGFR. Sanguinarine up-regulated NOX3 to elevate the reactive oxygen species (ROS) level, resulting in EGFR oxidation and degradation.^18^ Curcumin and the surviving inhibitor YM-155 degraded EGFR by inducing the ubiquitin-proteasomal pathway.^19,20^ Autophagic degradation of EGFR was involved in cancer cell death caused by arsenic and celastrol.^21,22^ Inhibiting canonical EGFR endocytosis by the clathrin inhibitor pitstop2 rerouted EGFR degradation to a macropinocytosis-mediated lysosomal pathway.^23^ The foregoing reports indicate that the mechanisms of EGFR endocytosis and degradation mediated by small molecules are complex and stimulus-dependent. Thus, the discovery of novel EGFR degraders and the exploration of EGFR degradation mechanisms are critical in the development of new strategies to control EGFR-positive NSCLC.

Up to now, few small-molecule ligands directly targeting EGFR and inducing EGFR degradation have been demonstrated. Here, we identify a new small-molecule EGFR ligand called DPBA, which functions as an EGFR degrader to inhibit the survival of EGFR-positive NSCLC. Specifically, DPBA binds to the EGFR extracellular domain (ECD) and induces flotillin-1-mediated EGFR endocytosis and lysosomal degradation without EGFR dimerization, phosphorylation, or ubiquitination. DPBA has the potential to be developed into a new drug for EGFR-positive NSCLC.

## Results

### DPBA reduces the viability of NSCLC cells by suppressing EGFR protein expression and the downstream pathways

**Fig. 1:**
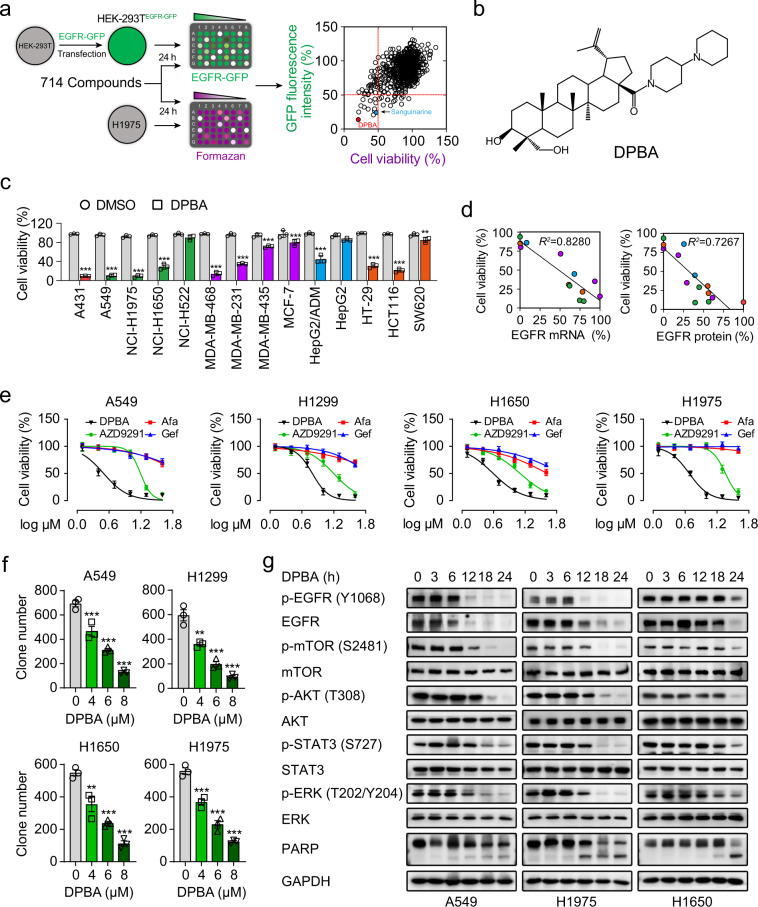
DPBA specifically reduces EGFR protein level and demonstrates potent anticancer effect towards NSCLC cells lines by suppressing EGFR protein level and the downstream pro-survival pathways. **a** EGFR degraders screening. **b** Chemical structure of DPBA. **c** DPBA showed potent anticancer effect towards EGFR-positive cancers. EGFR protein levels of A431, A549, H1975, H1650, H522, MDA-MB-468, MDA-MB-231, MDA-MB-435, MCF-7, HepG2/ADM, HepG2, HT-29, HCT116, and SW620 were measured by Western blot. All cell lines were treated with DPBA (5 μM) for 48 h. Cell viability was detected by the MTT assay. **P < 0.01, ***P < 0.001 vs. the control (DMSO) group, *n* = 3. **d** Correlation between viability and EGFR mRNA or protein level in aforementioned cell lines. **e** A549, H1299, H1650, and H1975 were treated with indicated concentrations of DPBA, gefitinib, afatinib, or AZD9291 for 24 h. Cell viability was measured by the MTT assay. **f** Cell colonies of A549, H1299, H1650, and H1975 treated with DPBA (4, 6, or 8 μM) were counted with ImagePro Plus. **P < 0.01, ***P < 0.001 vs. 0 μM, *n* = 3. **g** DPBA suppressed EGFR pro-survival pathway by reducing EGFR protein level. A549, H1975, and H1650 were treated with DPBA (6 μM) for the indicated times. Activation of EGFR pathway and cleavage of PARP were measured by Western blot

EGFR degraders were screened from >700 natural compounds and their derivatives (Supplementary Table 1). The screening process is described in the form of a flowchart in Fig. 1a. After test compounds’ treatment, green fluorescent protein (GFP) fluorescence intensity of HEK-239T^EGFR-GFP^ cells and viability of H1975 cells are determined, respectively. The well-defined EGFR degrader sanguinarine^18^ was the positive control (Fig. 1a). The most potent substance was compound **187**, which is a derivative of 23-hydroxybetulinic acid (23-HBA), namely DPBA (Fig. 1b), synthesized by our group previously.^24^ DPBA down-regulated only EGFR protein level among ErbB family members (Supplementary Fig. 1a), indicating that DPBA is an EGFR-specific inhibitor. We assessed the anticancer activity of DPBA (5 μM) on a panel of cancer cell lines in order to validate the association between the anticancer activity of DPBA and the EGFR levels. A431, A549, H1650, H1975, MDA-MB-468, and HCT116 with high EGFR expression levels were the most sensitive to DPBA (Fig. 1c). Cancer cell sensitivity to DPBA was positively correlated with the EGFR messenger RNA (mRNA) and protein levels (Fig. 1d).

We then determined the anticancer efficacy of DPBA against EGFR-positive NSCLC cell lines. The survival of A549 (EGFR WT), H1299 (EGFR WT), H1650 (del E736-A750), and H1975 (L858R/T790M) were suppressed to a greater extent by DPBA than by EGFR TKIs (gefitinib, afatinib, and AZD9291) (Fig. 1e). DPBA presented with lower cytotoxicity to human bronchial epithelial cells (BEAS-2B) and immortalized human keratinocytes (HaCaT) than NSCLC cell lines (Supplementary Fig. 1b). The colony formation assay confirmed the antiproliferation effect of DPBA on NSCLC cells (Fig. 1f). As the EGFR-driven pro-survival pathway is vital to tumour progression, we evaluated the effect of DPBA on EGFR downstream signalling pathway. DPBA significantly down-regulated EGFR protein in EGFR WT and EGFR mutant NSCLC cell lines in a time-dependent manner and did not increase EGFR phosphorylation (Fig. 1g). DPBA also reduced downstream p-mTOR, p-Akt, p-STAT3, and p-ERK phosphorylation and promoted the cleavage of the apoptotic marker PARP (poly (ADP-ribose) polymerase) (Fig. 1g). BEAS-AB and HaCaT cells treated with DPBA at the same concentrations presented with no EGFR protein inhibition (Supplementary Fig. 1c). Therefore, EGFR protein down-regulation blocked the EGFR-driven pro-survival pathway and conferred DPBA with anti-NSCLC activity.

### DPBA decreases EGFR protein levels by lysosomal degradation

**Fig. 2:**
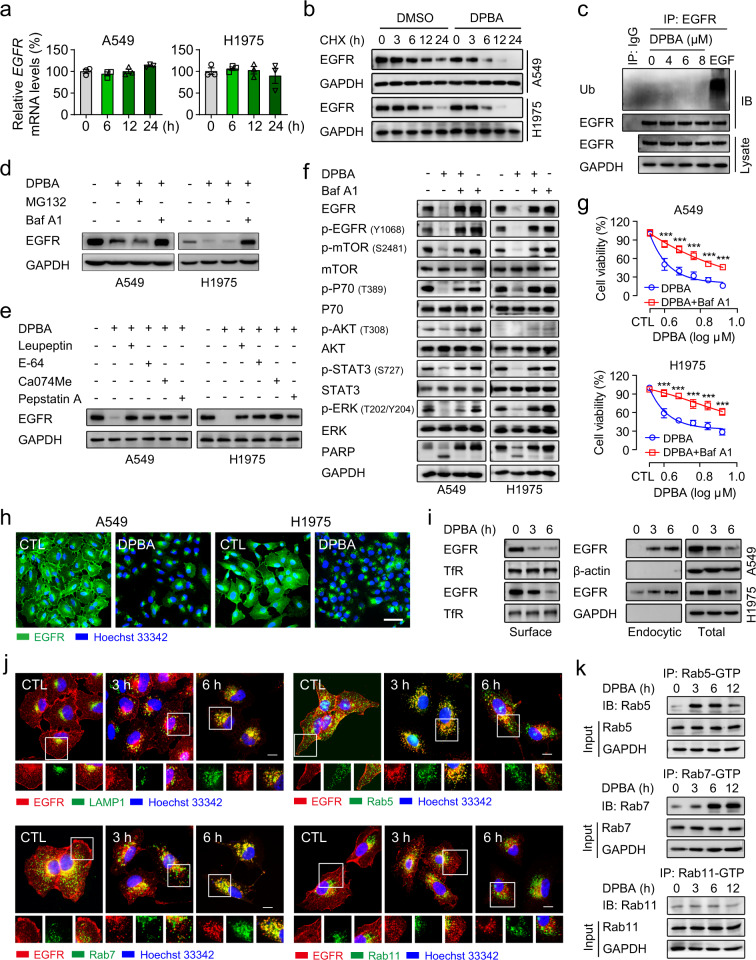
DPBA induces EGFR degradation in a lysosome-dependent manner. **a** DPBA did not reduce EGFR mRNA level. A549 and H1975 were treated with DPBA (6 μM) for 6, 12, and 24 h. EGFR mRNA level was measured by RT-PCR, *n* = 3. **b** DPBA enhanced EGFR half-time degradation. A549 and H1975 were treated with CHX (20 μM) in the presence or absence of DPBA (6 μM) for the indicated times. EGFR protein levels were measured by Western blot. **c** DPBA did not induce EGFR ubiquitination. A549 was treated with DPBA (4, 6, or 8 μM) for 3 h or EGF (100 ng/ml) for 5 min, and EGFR ubiquitination was measured by Western blot. **d** A549 and H1975 were treated with DPBA (6 μM) with or without MG132 (10 μM) or Baf A1 (200 nM) for 12 h. EGFR protein levels were measured by Western blot. **e** A549 and H1975 were treated with DPBA (6 μM) with or without leupeptin (100 μM), E-64 (200 μM), Ca074Me (10 μM), or pepstatin A (100 μM) for 12 h. EGFR protein levels were measured by Western blot. **f** A549 and H1975 were treated with DPBA (6 μM) in the presence or absence of Baf A1 (200 nM) for 24 h. Activation of EGFR pathway were measured by Western blot. **g** A549 and H1975 were treated with various concentrations of DPBA in the presence or absence of Baf A1 (200 nM) for 24 h. Cell viability was detected by the MTT assay, ***P < 0.001 vs. DPBA, *n* = 3. **h** DPBA induced EGFR perinuclear accumulation. A549 and H1975 were treated with DPBA (6 μM) for 6 h. EGFR sublocalisation was detected by immunofluorescence (magnification, ×200; scale bar, 50 μm). **i** DPBA induced surface EGFR endocytosis. A549 and H1975 were treated with DPBA (6 μM) for 3 and 6 h. Surface and intracellular EGFR were measured by biotinylation assay. TfR was the loading control for surface protein. **j** DPBA induced EGFR trafficking through endo-lysosome route. A549 was treated with DPBA (6 μM) for 3 and 6 h. Colocalisation between EGFR and LAMP1, Rab5, Rab7, or Rab11 was detected by immunofluorescence (magnification, ×630; scale bar, 10 μm). **k** DPBA activated Rab5 and Rab7, but not Rab11. A549 was treated with DPBA (6 μM) for 3, 6, and 12 h. Rab-GTP expression was measured with a Rab Activation Assay Kit

Protein expression may be reduced by the inhibition of de novo synthesis or by accelerated degradation. DPBA did not down-regulate EGFR mRNA (Fig. 2a). The cycloheximide (CHX) chase assay showed that the DPBA plus CHX treatment lowered the EGFR protein levels considerably more than the CHX treatment alone (Fig. 2b), indicating that DPBA down-regulates EGFR via protein degradation. Proteasomal and lysosomal degradation are two key protein degradation pathways. Here, DPBA did not induce EGFR ubiquitination (Fig. 2c). The lysosome inhibitor bafilomycin A1 (baf A1) but not the proteasome inhibitor MG132 reversed DPBA-induced EGFR degradation (Fig. 2d). This mechanism was confirmed with the lysosomal protease inhibitors leupeptin, E-64, Ca074Me, and pepstatin A (Fig. 2e). Baf A1 also reversed DPBA-induced inhibition of EGFR downstream signalling and cell death in A549 and H1975 (Fig. 2f, g). These data suggest that a lysosomal degradation pathway mediates DPBA-induced EGFR protein decrease.

### Rab5 and Rab7 activation is involved in DPBA-induced endo-lysosomal trafficking of EGFR

The induction of endocytosis is a key step in EGFR degradation. We observed DPBA-induced perinuclear EGFR cluster formation (Fig. 2h). Next, we conducted a surface biotinylation assay to establish that the perinuclear clusters were derived from intracellular or membrane-bound EGFR. As shown in Fig. 2i, surface EGFR was significantly decreased, whereas plasma membrane-bound EGFR labelled with NHS-SS-biotin had substantially increased in the cytoplasm after DPBA treatment, indicating that DPBA induces surface EGFR endocytosis. Endocytic EGFR may be transported to lysosomes via early and late endosomes, enter recycling endosomes and returned to the plasma membranes, or delivered to the Golgi apparatus, endoplasmic reticulum, mitochondria or nuclei, depending on the stimulating factors.^25^ DPBA did not induce EGFR colocalisation with autophagosomes (LC-3-positive), mitochondria (TOM20-positive), Golgi apparatus (GM130-positive), or endoplasmic reticulum (calnexin-positive) (Supplementary Fig. 2a). However, DPBA-induced endocytic EGFR colocalised with Rab5 after 3 h and entered the late endosomes (Rab7-positive) and lysosomes (LAMP1-positive) after 6 h. No endocytic EGFR colocalisation with recycling endosomes (Rab11-positive) was detected (Fig. 2j). Consistently, DPBA increased the Rab5-GTP and Rab7-GTP levels, but not the Rab11-GTP level (Fig. 2k). Collectively, these data demonstrate that DPBA-induced endocytic EGFR get the label of lysosome degradation rather than being recycled to the plasma membranes.

### DPBA induces EGFR endocytosis via a clathrin-independent lipid raft

**Fig. 3:**
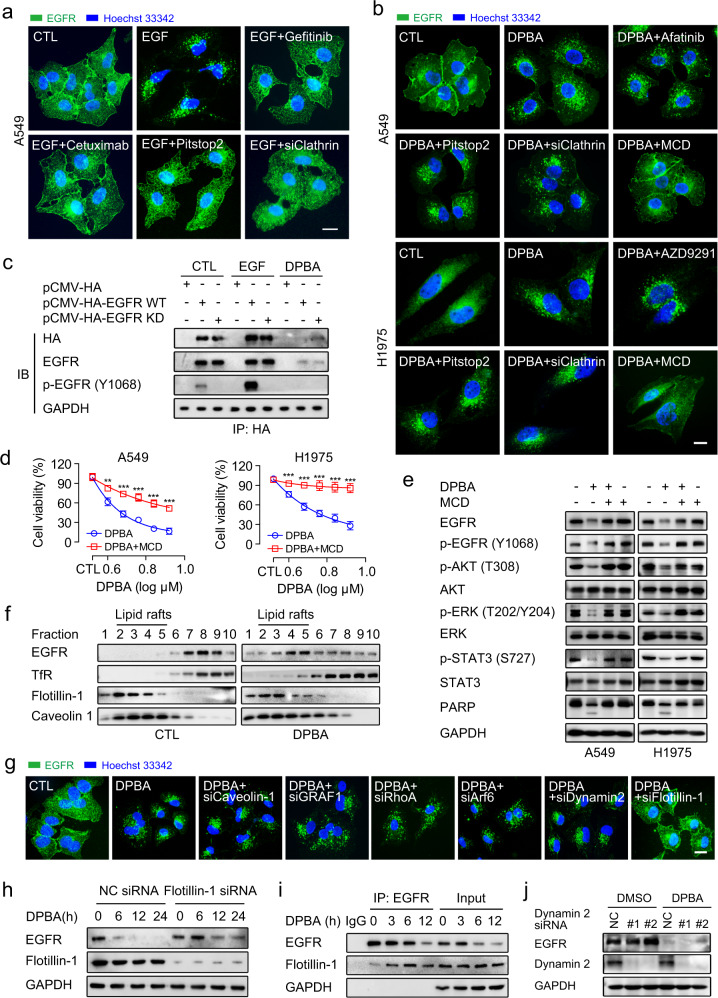
DPBA-induced EGFR endocytosis is clathrin-independent but lipid raft-mediated. **a** EGF-induced EGFR endocytosis was impaired by inhibition of EGFR kinase activity or clathrin. A549 was treated with EGF (20 ng/ml) in the presence or absence of gefitinib (10 μM), cetuximab (5 μg/ml), pitstop2 (5 μM), or clathrin siRNA for 30 min. EGFR endocytosis was observed by immunofluorescence (magnification, ×630; scale bar, 10 μm). **b** DPBA-induced EGFR endocytosis was blocked by a lipid raft inhibitor MCD. A549 and H1975 were treated with DPBA (6 μM) in the presence or absence of afatinib (10 μM), AZD9291 (10 μM), pitstop2 (5 μM), MCD (1 mg/ml), or clathrin siRNA for 6 h. EGFR endocytosis was observed by immunofluorescence (magnification, ×630; scale bar, 10 μm). **c** DPBA degraded both EGFR WT and EGFR KD. HEK-293T transfected with pCMV-HA, pCMV-HA EGFR WT, or pCMV-HA EGFR KD was treated with EGF (20 ng/ml) for 30 min or DPBA (6 μM) for 24 h. Exogenous EGFR was subjected to pull-down with anti-HA antibody. EGFR and p-EGFR (Y1068) levels were measured by Western blot. **d** A549 and H1975 were treated with indicated concentrations of DPBA in the presence or absence of MCD (1 mg/ml) for 24 h. Cell viability was measured by MTT assay, **P < 0.01, ***P < 0.001 vs. DPBA, *n* = 3. **e** A549 and H1975 were treated with DPBA (6 μM) with or without MCD (1 mg/ml) for 24 h. Total EGFR, p-EGFR (Y1068), Akt, p-Akt (T308), ERK, p-ERK (T202/Y204), STAT3, p-STAT3 (S727), and PARP expression levels were measured by Western blot. **f** DPBA induced EGFR accumulation in lipid rafts. A549 was treated with DPBA (6 μM) for 3 h. EGFR distributions in lipid rafts were detected by density gradient centrifugation and Western blot. TfR was a non-lipid raft marker while caveolin-1 and flotillin-1 were lipid raft markers. **g** Flotillin-1 knockdown blocked DPBA-induced EGFR endocytosis. A549 was transfected with dynamin 2 siRNA, flotillin-1 siRNA, caveolin-1 siRNA, GRAF1 siRNA, RhoA siRNA, and Arf6 siRNA for 48 h, followed by DPBA treatment (6 μM) for 6 h. EGFR endocytosis was observed by immunofluorescence (magnification, ×630; scale bar, 10 μm). **h** Flotillin-1 knockdown inhibited DPBA-induced EGFR degradation. A549 was transfected with flotillin-1 siRNA (100 nM) for 48 h, followed by DPBA treatment (6 μM) for 24 h. EGFR degradation was detected by Western blot. **i** DPBA enhanced interaction of EGFR and flotillin-1. A549 was treated with DPBA (6 μM) for 3, 6, and 12 h. Interactions between EGFR and flotillin-1 were detected by co-IP. **j** A549 was transfected with dynamin 2 siRNA (100 nM) for 48 h, followed by DPBA treatment (6 μM) for 24 h. EGFR degradation was detected by Western blot

EGF-induced EGFR endocytosis is primarily mediated by clathrin and dependent on tyrosine kinase activity.^26,27^ As expected, the suppression of EGFR activation with gefitinib or cetuximab or the inhibition of clathrin with pitstop2 or clathrin siRNA blocked EGF-induced EGFR endocytosis (Fig. 3a, Supplementary Fig. 2b). However, gefitinib, AZD9291, pitstop2, or clathrin siRNA did not suppress DPBA-induced EGFR endocytosis (Fig. 3b). DPBA treatment also degraded kinase-dead EGFR (Fig. 3c). These data clearly show that DPBA-induced EGFR endocytosis is clathrin independent and does not require EGFR tyrosine kinase activity. However, EGFR still has serine/threonine phosphorylation sites, which also play important roles in EGFR regulation, including EGFR endocytosis.^28,29^ We found that DPBA had no effect on EGFR serine/threonine phosphorylation level, indicating that Ser/Thr phosphorylation may not be involved in DPBA-induced EGFR endocytosis (Supplementary Fig. 2c).

Clathrin-independent EGFR endocytosis is achieved by a lipid raft-dependent process or macropinocytosis.^23,30^ The macropinocytosis inhibitor amiloride blocked fluorescein isothiocyanate-dextran (FITC-dextran) uptake, but failed to block DPBA-induced EGFR endocytosis (Supplementary Fig. 2d). Here, the cholesterol extractor methyl-β-cyclodextrin (MCD) substantially blocked the EGFR endocytosis (Fig. 3b), downstream pathway down-regulation, and cell death induced by DPBA (Fig. 3d, e). As cholesterol is an important lipid raft constituent, the foregoing results indicate that DPBA-induced EGFR endocytosis is mediated by lipid rafts. It was further confirmed by the fact that DPBA induced a significant EGFR residence in the lipid raft domain (Fig. 3f). Taken together, our results demonstrate that the lipid raft microdomain serves as an organizational platform for the initiation of DPBA-induced EGFR endocytosis.

### DPBA-induced EGFR endocytosis depends on flotillin-1

Lipid raft-dependent endocytosis is mediated either by caveolin or flotillin or by small guanosine triphosphatases, such as GRAF1, Arf6, or RhoA.^31^ Here, only flotillin-1 knockdown blocked DPBA-induced EGFR endocytosis and degradation (Fig. 3g, h, Supplementary Fig. 2e). In flotillin-1-mediated endocytosis, flotillin-1 is recruited to the surface cargo and forms pre-endocytic clusters.^32^ DPBA treatment also enhanced the interaction between flotillin-1 and EGFR (Fig. 3i). As dynamin 2 catalyses endocytic vesicle scission,^31^ we investigated the role of dynamin 2 in DPBA-induced EGFR endocytosis and degradation. Dynamin 2 siRNA did not recover DPBA-induced EGFR endocytosis or degradation (Fig. 3g, j). This mechanism was confirmed with the potent dynamin 2 inhibitor dyngo-4a (Supplementary Fig. 2f). Thus, flotillin-1 is a key moderator in DPBA-induced EGFR endocytosis and the latter is independent of dynamin 2.

### DPBA directly binds to EGFR ECD and triggers EGFR degradation

**Fig. 4:**
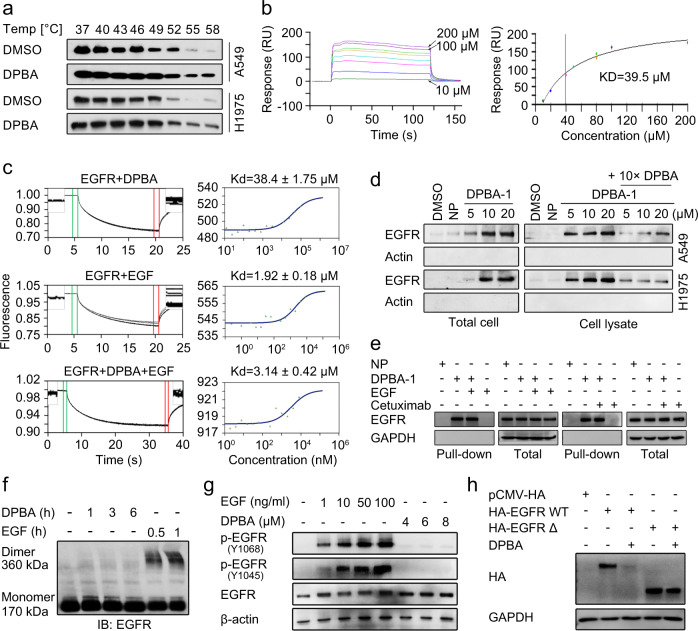
DPBA directly binds to EGFR ECD. **a** DPBA thermally stabilized EGFR in cellular level. A549 and H1975 were treated with DPBA (6 μM) for 3 h. Lysates were divided into eight fractions, followed by heating to indicated temperatures. Soluble EGFR was detected by Western blot. Interaction between DPBA (200, 100, 80, 80, 50, 40, 20, and 10 μM) and EGFR ECD was measured by BIAcore (**b**) or MST (**c**). **d** DPBA-1 dose-dependently interacted with EGFR in both total cell and cell lysate. In the total cell assay, A549 and H1975 were treated with NP (20 μM) or indicated concentrations of DPBA-1 for 1 h. In the cell lysate assay, A549 and H1975 were lysed, followed by indicated treatment. Interactions between EGFR and DPBA-1 were detected by pull-down assay. **e** EGF or cetuximab did not block the interaction of EGFR and DPBA-1. A549 was treated with DPBA-1 (10 μM) in the presence or absence of EGF (10 ng/ml) or cetuximab (5 μg/ml) for 1 h. Interactions between EGFR and DPBA-1 were detected by pull-down assay. **f** DPBA did not induce EGFR dimerization. A549 was treated with DPBA (6 μM) for 1 h, 3 h, and 6 h, or with EGF (200 ng/ml) for 0.5 and 1 h. EGFR dimerization was detected by Western blot. **g** A549 was treated with indicated concentrations of EGF or DPBA for 30 min. Total EGFR, p-EGFR (Y1068), and p-EGFR (Y1045) were measured by Western blot. **h** Deletion of EGFR ECD impaired DPBA-induced EGFR degradation. HEK-293T transfected with pCMV-HA, HA EGFR WT, or HA EGFR Δ was treated with DPBA (6 μM) for 24 h, and HA expression level was measured by Western blot

As the ligand EGF binds to EGFR and induces EGFR activation and degradation, we attempted to determine whether DPBA has a similar mode of action. We used cellular thermal shift assay to test the cell-level interaction between DPBA and EGFR. Relative to the control, EGFR showed thermal shifts in the presence of DPBA at the denaturation temperature range of 37–58 °C (Fig. 4a), indicating that DPBA may directly bind to EGFR and thermally stabilize it in vivo. We then performed BIAcore kinetics and microscale thermophoresis (MST) analyses on binding between recombinant EGFR ECD and DPBA, and EGF was the positive control. The BIAcore assay showed that DPBA bound to EGFR ECD, and the association constant (*K*_d_) was 39.5 μM (Fig. 4b, Supplementary Fig. 3a). However, the curve disclosed distinct binding kinetics between DPBA and EGF, indicating that each has its unique binding characteristic with EGFR. The MST assay revealed that DPBA interacted with EGFR ECD in a dose-dependent manner and *K*_d_ = 38.4 ± 1.75 μM, whereas *K*_d_ = 1.92 ± 0.18 μM for EGF. However, the presence of DPBA did not affect the interaction between EGFR ECD and EGF as the *K*_d_ did not markedly change (3.14 ± 0.42 μM) (Fig. 4c). These data imply that DPBA and EGF have different binding sites for EGFR. DPBA did not interact with the intracellular domain (ICD) of EGFR, or the ECD of Her2, Her3, or Her4 (Supplementary Fig. 3b). Therefore, DPBA specifically binds to EGFR ECD. The parent compound of DPBA (23-HBA) did not bind to EGFR ECD and had no effect on the EGFR protein level (Supplementary Fig. 3b, c), suggesting that the dipiperidine group may be an absolute requirement for the interaction between DPBA and EGFR.

To further confirm the interaction between DPBA and EGFR, we synthesized the probe DPBA-1, using a photoaffinity-labelling technique by introducing a tetrazole-containing base with an alkynyl terminal to the inactive C-23 site. A negative probe (NP) was used as the negative control (Supplementary Fig. 3d). The introduction of the tetrazole-containing base had no influence on cytotoxicity and EGFR degradation of DPBA (Supplementary Fig. 3e, f). The pull-down assay showed that DPBA-1 interacted with EGFR in a dose-dependent manner in the intact cells and cell lysates. Excess DPBA (10×) significantly inhibited the interaction between DPBA-1 and EGFR, indicating that both competed for the same site (Fig. 4d). Neither EGF nor cetuximab interfered with the interaction between EGFR and DPBA-1 (Fig. 4e). This observation suggests that DPBA binds to sites different from those of EGF and cetuximab. EGF binding to EGFR induced EGFR dimerization and activation, whereas DPBA did neither (Fig. 4f, g). Thus, DPBA and EGF differ in terms of binding sites and mechanisms. We next checked whether EGFR ECD binding was crucial for DPBA-induced EGFR degradation. As shown in Fig. 4h, EGFR ECD deletion (del 25–645) blocked DPBA-induced EGFR degradation. Collectively, these results indicate that DPBA specifically binds to EGFR ECD to trigger EGFR degradation.

### DPBA inhibits EGFR WT and EGFR mutant NSCLC growth in vivo

**Fig. 5:**
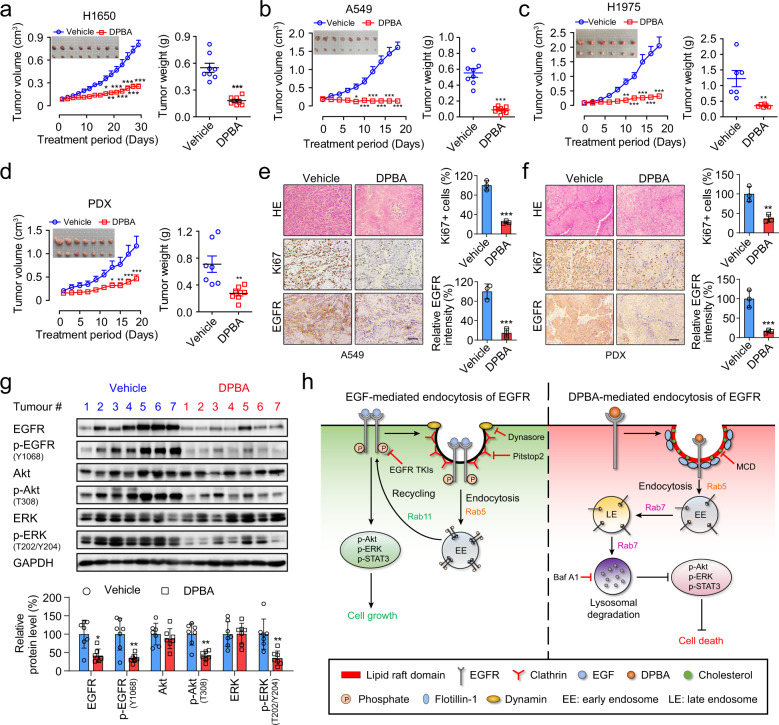
DPBA inhibits NSCLC growth in vivo. Tumour photos, tumour volume curves, tumour weights, and body weight curves for H1650 (**a**), A549 (**b**), H1975 (**c**), and primary lung PDX (**d**). *P < 0.05; **P < 0.01; ***P < 0.001 vs. the vehicle group. H&E staining and immunohistochemistry (IHC) staining for Ki67 and EGFR in A549 (**e**) and PDX (**f**) tumour tissues. Scale bar, 200 μm; **P < 0.01; ***P < 0.001 vs. the vehicle group, *n* = 3. **g** DPBA significantly suppressed EGFR pathway in PDX tumour tissues. *P < 0.05; **P < 0.01 vs. the vehicle group. **h** Schematic representation of the anticancer mechanism of EGFR small-molecule ligand DPBA

To examine the anti-NSCLC activity of DPBA in vivo, we established the tumour xenografts models of A549, H1650, and H1975 cells, and a xenograft derived from a patient with primary EGFR-positive lung cancer (Supplementary Fig. 4a). Mice treated with DPBA (25 mg/kg) presented with considerable tumour growth inhibition, but there was no change in body weight (Fig. 5a–d, Supplementary Fig. 4b, c). Haematoxylin–eosin (H&E) staining revealed that DPBA induced substantial cell death in the tumour sections. Ki67, a marker of proliferation and EGFR expression levels considerably decreased after DPBA treatment (Fig. 5e, f). The EGFR pathway was markedly repressed in the DPBA-treated patient-derived xenograft (PDX) model (Fig. 5g). To assess DPBA toxicity, we collected mouse blood and organs for serum biochemistry, routine blood analyses, and pathological examination, respectively. As shown in Supplementary Fig. 4d, the white blood cells, red blood cells, platelets, and haemoglobin counts did not significantly change in response to DPBA exposure. Lactate dehydrogenase, creatine kinase, alanine transaminase, aspartate aminotransferase, creatinine, blood urea nitrogen, and spleen weight were not significantly altered by DPBA treatment. H&E staining of the kidney, spleen, liver, and heart revealed that DPBA had no significant toxic effects on any of them (Supplementary Fig. 4e). Overall, DPBA induced EGFR degradation and markedly inhibited the growth of EGFR-positive NSCLC xenografts with negligible toxicity.

## Discussion

Due to the inevitable acquired resistance and kinase-independent functions of EGFR, targeting EGFR degradation by small molecules is a promising cancer treatment strategy.^33^ Here, we identified the 23-HBA derivative DPBA that induces EGFR degradation by directly binding to EGFR ECD, blocking downstream pathways and thereby suppressing NSCLC growth. Mechanistically, DPBA binding to EGFR ECD differs from that between EGFR and TKIs. Thus, DPBA induces EGFR degradation and cell death regardless of the mutations in the ICD, such as del E746-A750, L858R, and T790M. Unlike EGF or a reported small-molecule EGFR ligand NSC228155,^34^ DPBA neither induces EGFR dimerization nor activates EGFR and its downstream pathways. In this way, it avoids the pro-survival effects of EGFR activation. Moreover, DPBA does not alter EGFR expression in normal human cells. DPBA potently inhibits the growth of several EGFR-positive NSCLC xenografts and has negligible toxicity. Overall, DPBA is a novel EGFR degrader that could potentially treat both EGFR WT and EGFR mutant NSCLC.

EGFR ICD initiates the EGFR signalling pathway and is a major EGFR target. EGFR TKIs and PROTAC EGFR degraders target this region. EGFR degradation was also achieved by targeting the allosteric site of the EGFR kinase domain by weak reversible EGFR inhibitors without connecting of proteasomal degradation tags to EGFR TKIs.^35^ The aforementioned strategies are effective and specific to achieve EGFR degradation. However, most EGFR mutations focus on the kinase domain.^36^ Thus, inducing EGFR degradation by targeting the kinase region may not avoid EGFR mutations. An alternative approach to EGFR degradation is to target the ECD. It has been reported recently that T315 initiates EGFR degradation by binding the ECD. Its action depends on Y1045 phosphorylation and CBL recruitment. CBL is an E3 ubiquitin-protein ligase that drives EGFR proteasomal degradation.^37^ This process aligns with the conventional EGF-induced EGFR degradation mechanism and indicates that T315 may function in a way similar to that of EGF.^38^ DPBA did not induce EGFR phosphorylation, dimerization, or ubiquitination, and EGFR TKIs and MG132 did not rescue EGFR degradation. Thus, DPBA regulates EGFR in a heretofore unknown manner. Furthermore, we performed de novo liquid chromatography mass spectrometry (LC-MS) sequencing analysis using a photoaffinity-labelled probe DPBA-1, and identified three potential sites, D166, D303, and D321. The alkaline dipiperidine group in DPBA may be important for the binding to the acidic amino acids of EGFR. Site-directed mutagenesis experiments need be further conducted on EGFR to localize its key binding sites. Besides, the crystalline structure of EGFR bound to DPBA will be helpful to reveal the binding sites and conformation of EGFR with DPBA. Given that DPBA-induced EGFR degradation is resistant to EGFR TKIs, EGFR kinase-independent functions may be involved. These non-canonical EGFR functions are mostly achieved by interacting with other chaperone proteins, such as HSP90, CDC34, and SGLT1. These interaction levels are higher in tumour cells than in normal cells.^10,39–44^ DPBA may destabilize EGFR through interrupting these interactions. This may partially explain why DPBA showed specific toxicity towards tumour cells. Interaction proteomics is required to analyse potential interaction partners and reveal the degradation mechanism of EGFR in tumour cells upon DPBA binding. It has been reported that EGFR dimerization not only induces autophosphorylation but also maintains EGFR stability. Impairing EGFR dimerization by a specific peptide can induce EGFR degradation.^45^ Whether DPBA has a similar potency needs further investigation. Nevertheless, the discovery of DPBA as a novel EGFR ligand underscores the fact that EGFR may be degraded by targeting EGFR ECD. Elucidation of the binding and interaction mechanisms between DPBA and EGFR will help guide the design of new EGFR degraders that selectively target EGFR ECD.

The endocytic mechanism and post-endocytic fates of EGFR are complex and stimulus-dependent.^25,26^ Canonical EGF-induced EGFR endocytosis is regulated by both clathrin-mediated endocytosis (CME) and non-CME (NCE). CME is activated at all EGF concentrations, whereas NCE is induced only at high EGF concentrations wherein the receptor is internalized via lipid rafts.^46^ Ligand-independent EGFR endocytosis has also been detected in response to various stimuli such as chemotherapeutic agents, ultraviolet, EGFR TKIs, EGFR monoclonal antibodies and ROS.^25^ EGFR TKIs induced the internalization of inactivated EGFR and endosomal arrest, which is considered an innate TKI resistance mechanism.^47^ Ultraviolet (UV) or cisplatin-induced clathrin-mediated EGFR internalization, which entails serine and threonine phosphorylation catalysed by p38 kinase.^48,49^ Cetuximab stimulated caveolin-mediated EGFR endocytosis that was independent of tyrosine kinase activity but mediated by p38.^50–52^ CHX-induced EGFR endocytosis was also p38-dependent, but tyrosine kinase-independent.^53^ It seems that EGFR tyrosine kinase activity is expendable, whereas p38 plays a key role in EGFR endocytosis. DPBA-induced EGFR endocytosis is also tyrosine kinase independent, but the p38 inhibitor SB203580 did not block EGFR endocytosis (data not shown). This finding is consistent with that of a previous study, which showed that p38-mediated endocytic EGFR is recycled back to the plasma membrane.^54^ Here, we revealed a novel DPBA-initiated EGFR endocytosis mechanism that is mediated by a flotillin-dependent lipid raft and does not involve EGFR dimerization, phosphorylation, and ubiquitination. This unique mechanism enables DPBA to serve as a new chemical probe in the ongoing exploration of EGFR endocytosis and trafficking.

23-HBA, a lupane-type pentacyclic triterpene extracted from the Chinese medicinal herb *Pulsatilla chinensis* by our group, has been found to exhibit anti-tumour activity in vitro and in vivo. In order to improve its biological activity, we carried out structural modifications and obtained a series of derivatives with improved activities.^24^ The anti-tumour mechanisms of 23-HBA or its derivatives mainly involve mitochondrial ROS burst, mitochondrial membrane potential depolarization, non-classical mitochondrial autophagy, telomerase activity inhibition, and multidrug resistance reversal.^55–58^ So far, there have been no reports on 23-HBA or its derivatives, whose modes of action resembles that of DPBA. Our study sheds a new insight into the molecular mechanism of 23-HBA and its derivatives.

The present study identifies one 23-HBA derivative, DPBA, a promising anticancer drug candidate that binds to EGFR ECD and promotes EGFR endocytosis and lysosomal degradation in the treatment of EGFR-positive NSCLC (Fig. 5h). Here, we unveiled a new class of small-molecule EGFR degraders directly targeting EGFR ECD. In this way, we provide a strategy to inhibit EGFR kinase-independent functions and suppress innate or acquried EGFR TKI-mediated NSCLC resistance.

## Materials and methods

### Cell culture

A431, A549, NCI-H1299, NCI-H1650, NCI-H1975, NCI-H522, MDA-MB-231, MDA-MB-435, MDA-MB-453, MDA-MB-468, MCF-7, HepG2, HT-29, HCT116, SW620, HEK-293T, and BEAS-2B were purchased from the American Type Culture Collection (Manassas, VA, USA). HaCaT was obtained from Beina Chuanglian Biotechnology Research Institute (Beijing, China). HepG2/ADM cells were generously provided by Prof. Kwok-Pui Fung (Chinese University of Hong Kong, Hong Kong, China). H1299, H1650, and H1975 were cultured in RPMI-1640 medium (Thermo Fisher Scientific, Waltham, MA, USA) supplemented with 10% (v/v) foetal bovine serum (FBS, Thermo Fisher Scientific) and 1% (v/v) penicillin–streptomycin (PS, Thermo Fisher Scientific). All other cell lines were cultured in Dulbecco’s modified Eagle’s medium supplemented with 10% (v/v) FBS and 1% (v/v) PS. Cells were maintained at 37 °C in a humidified atmosphere incubator with 5% CO_2_. Cell line authentication and detection of mycoplasma contamination were performed before usage.

### Reagents and antibodies

DPBA (98% purity) was synthesized as described previously.^24^ DAPI (4′,6-diamidino-2-phenylindole), CHX, leupeptin, E-64, Ca074Me, pepstatin A, pitstop2, MCD, FITC-dextran, Tris (2-carboxyethyl) phosphine hydrochloride (TCEP), Tris [(1-benzyl-1*H*-1,2,3-triazol-4-yl) methyl] amine (TBTA), Biotin-N_3_ and Azide-fluor 545 were purchased from Sigma-Aldrich (St. Louis, MO, USA). Hoechst 33342, BS3 (bis (sulfosuccinimidyl) suberate), EZ-Link Sulfo-NHS-SS-Biotin, biotinamidohexanoic acid *N*-hydroxysuccinimide ester, Pierce™ Avidin Agarose, a BCA Protein Assay Kit, and a DAB Kit were obtained from Thermo Fisher Scientific. Gefitinib, afatinib, AZD9291, MG132, Baf A1, Amiloride HCl, and dyngo-4a were purchased from Selleck (Houston, TX, USA). Laemmli sample buffer (2×) and an ECL Chemiluminescence Detection Kit were purchased from Bio-Rad (Hercules, CA, USA). Matrigel was obtained from BD Biosciences (San Jose, CA, USA). A Total RNA Kit was purchased from OMEGA BIO-TEK (Norcross, GA, USA). SYBR Green I Master and a Transcriptor First Strand cDNA Synthesis Kit was obtained from Roche (Mannheim, Germany). Antibodies against PARP, cleaved PARP, EGFR (rabbit monoclonal), Her2, Her3, Her4, p-EGFR (Y1068), p-EGFR (Y1045), mTOR, p-mTOR (S2481), STAT3, p-STAT3 (S727), Akt, p-Akt (T308), ERK, p-ERK (T202/204), phospho-threonine, transferrin receptor 1 (TfR), Rab5, Rab7, Rab11, LC-3, flotillin-1, caveolin-1, GRAF1, Arf6, Ki67, β-actin, GAPDH, anti-rabbit IgG, and anti-mouse IgG were obtained from Cell Signaling Technology (Beverly, MA, USA). Antibodies against EGFR (mouse monoclonal), dynamin 2 and RhoA were obtained from Abcam (Cambridge, MA, USA). Phospho-serine antibody was purchased from Merck Millipore (Darmstadt, Germany). All other reagents were purchased from Sigma-Aldrich.

### Plasmid construction and transfection

EGFR-GFP was obtained from Addgene (#32751). pCMV-HA EGFR was constructed by cloning EGFR WT complementary DNA (cDNA) into the pCMV-HA vector. pCMV-HA EGFR KD (K745A, kinase dead) was generated from pCMV-HA EGFR using KOD-Plus Mutagenesis Kit (Toyobo, Japan). Primers for EGFR KD were as follows: ATCGCGGAATTAAGAGAAGCAACAT (forward); AGCGACGGGAATTTTAACTTTCTCA (reverse). EGFR ECD deletion (del 25–645) cDNA was synthesized by Beijing Genomics Institute (Beijing, China) and cloned into the pCMV-HA vector to generate pCMV-HA EGFR Δ. All these plasmids were constructed by using the restriction sites *Sal*l and *Not*l. Transfections were performed by using Lipofectamine 3000 reagent according to the manufacturer’s protocol.

### Cell viability assay

Cells with indicated treatment were incubated with MTT (3-(4,5-dimethylthiazol-2-yl)-2,5-diphenyl-2*H*-tetrazolium bromide; 5 mg/ml) for 4 h. Dimethyl sulfoxide (DMSO) were added to solubilize the formazan crystals, and the absorbance was measured at 595 nm using a microplate reader (Beckman Coulter, Brea, CA, USA). Cell viability was calculated as a percentage of the vehicle control group treated with a medium containing 0.2% DMSO.

### Western blot

Cells were lysed with RIPA lysis buffer containing protease inhibitor cocktail. Thirty micrograms of total protein was separated on sodium dodecyl sulphate-polyacrylamide gel electrophoresis (SDS-PAGE) gels and then transferred onto polyvinylidene fluoride membranes. The levels of indicated proteins were blotted by incubating with primary antibodies overnight at 4 °C, followed by incubation with secondary antibodies for 1 h at room temperature (RT). Immunoreactive proteins were visualized using an ECL Chemiluminescence Detection Kit.

### Colony formation assay

Cells treated with indicated concentrations of DPBA for 24 h were trypsinized and seeded in 6-well plates as a density of 0.8 × 10^3^ cells/well and cultured for 10 days. At the end, cells were fixed with 4% paraformaldehyde (PFA) and stained with a 0.1% crystal violet solution. Cell colonies were photographed by a CKX41 inverted microscope (Olympus, Japan) and counted using ImagePro Plus v. 6.0 (Media Cybernetics Inc., Rockville, MD, USA).

### Reverse transcription polymerase chain reaction (RT-PCR) assay

Two micrograms of total RNA were transformed to cDNA by a Transcriptor First Strand cDNA Synthesis Kit. RT-PCR was performed by mixing 10 μl SYBR Green I Master, 0.5 μM forward primer, 0.5 μM reverse primer, 2 μl cDNA, and 6 μl distilled water per sample. The PCR products were quantified with a LightCycler 480 PCR system (Roche, Mannheim, Germany). The primers used for PCR were synthesized by Beijing Genomics Institute and the sequences for EGFR were: 5′-CCTGGTCTGGAAGTACGCAG-3′ and 5′-CGATGGACGGGATCTTAGGC-3′.

### Immunofluorescence assay

Cells with indicated treatments were fixed in 4% PFA and blocked in 5% bovine serum albumin containing 0.4% Triton X-100. Then, the cells were incubated using the indicated primary antibody at 4 °C overnight, followed by a fluorescent secondary antibody for 1 h at RT and stained with 5 μg/ml of DAPI for 5 min. The cellular fluorescence was photographed by a Zeiss AX10 microscope (Carl Zeiss, Göttingen, Germany).

### EGFR endocytosis assay

Surface and endocytic EGFR were localized by biotinylation assay.^59^ For the surface EGFR, the cells were biotinylated with 0.5 mg/ml NHS-SS-biotin for 30 min at 4 °C, washed with ice-cold phosphate-buffered saline (PBS), and blocked with 50 mM glycine for 20 min. For the endocytic EGFR, the cells were biotinylated with NHS-SS-biotin and blocked with glycine before the DPBA treatment. Surface NHS-SS-biotin EGFR was reduced with 50 mM glutathione in 90 mM NaCl, 1 mM MgCl_2_, 0.1 mM CaCl_2_, 60 mM NaOH, and 10% (v/v) FBS for 30 min. The cells were then lysed and the biotinylated EGFR was precipitated with avidin agarose beads for 2 h at 4 °C. The EGFR was detected by Western blot.

### Rab-GTP activity analysis

Activation of Rab5, Rab7, and Rab11 were analysed with Rab5, Rab7, and Rab11 Activation Assay Kits (NewEast Biosciences, King of Prussia, PA, USA). Briefly, 500 μg total protein lysed with 1× lysis buffer per sample were used in a Rab-GTP pull-down assay. Each sample was combined with anti-Rab-GTP monoclonal antibody and incubated at 4 °C overnight. Protein A/G agarose beads were used to capture the antibodies. After 1 h incubation at 4 °C, the beads were boiled in 2× loading buffer for 5 min. The supernatant was used for Western blot to detect Rab-GTP activity using Rab polyclonal antibody.

### siRNA transfection assay

Cells were transfected with control siRNA duplexes or specific siRNA duplexes with indicated targets using Lipofectamine 3000. After transfection for 48 h, cells were exposed to indicated treatment, and the expression levels of specific proteins were measured by Western blot. siRNAs were synthesized by GenePharma (Shanghai, China) and the sequences were shown in Supplementary Table 2.

### Preparation of detergent-free lipid rafts

Detergent-free lipid rafts were prepared as described previously.^60^ After washing with ice-cold PBS, cells were resuspended in 0.5 ml lysis buffer (20 mM Tis-HCl, 250 mM sucrose, 1 mM CaCl_2_, 1 mM MgCl_2_, pH = 7.8) with protease inhibitor cocktail and lysed by passing through a 22-G needle 40 times. The post-nuclear supernatant was collected by centrifugation at 1000 × *g* for 10 min, while the precipitated pellet was lysed again in the same way in 0.5 ml lysis buffer and the post-nuclear supernatant was combined with the first. One millilitre of lysis buffer with 50% (v/v) OptiPrep was added to the supernatants and transferred to an ultra-centrifuge tube. Three millilitres of 0–20% gradient OptiPrep in lysis buffer was placed on the top of the mixture. After centrifugation at 25,000 r.p.m. for 90 min in a Beckman ultra-centrifuge, 10 fractions were collected from top to bottom of the gradients. EGFR, TfR, flotillin-1, and caveolin-1 in different fractions were analysed by Western blot.

### Cetuximab biotinylation

Cetuximab biotinylation was performed as described previously.^61^ In brief, glycine was removed from Erbitux by ultrafiltration in 0.9% NaCl. Cetuximab and biotinamidohexanoic acid *N*-hydroxysuccinimide ester dissolved in DMSO (1 mg/ml) were mixed in the ratio of 1:2. Two micrograms of cetuximab without glycine was incubated with 12 μg biotinamidohexanoic acid *N*-hydroxysuccinimide ester at 4 °C overnight. Ten microlitres of 10× PBS was added to adjust pH to 7.0. The process was stopped by adding 20 μl 2 M Tris and incubated at RT for 60 min. Excess biotinamidohexanoic acid *N*-hydroxysuccinimide was removed by ultrafiltration.

### Co-immunoprecipitation

Cells were lysed in immunoprecipitation buffer (Thermo Fisher) with protease inhibitor cocktail. EGFR was immunoprecipitated by incubating with biotinylated cetuximab at 4 °C overnight, followed by incubation with avidin agarose beads at RT for 2 h. Immune complexes were washed five times with immunoprecipitation buffer, then eluted by boiling in 2× loading buffer for 5 min. The expression levels of interacting proteins were detected by Western blot.

### Cellular thermal shift assay

Cells treated with DPBA were disrupted by liquid nitrogen, then centrifuged at 12,000 r.p.m. for 15 min to get total protein extracts. The proteins were divided into eight fractions and denatured at different temperatures for 10 min. Denatured proteins were precipitated by centrifugation at 12,000 r.p.m. for 10 min. Supernatant undenatured EGFR was detected by Western blot.

### BIAcore

Recombinant human EGFR ECD (Sino Biological, Beijing, China) was directly coupled to CM-5 chip’s (GE Healthcare Life Sciences, Marlborough, MA, USA) different channel according to the isoelectric point of EGFR protein. The interaction between EGFR and DPBA was measured by a BIAcore S200 (GE Healthcare Life Sciences). Affinity curve and kinetic curve were finally obtained using Biacore S200 Evaluation Software. EGF was used as a positive control.

### Microscale thermophoresis

Recombinant human EGFR ECD, EGFR ICD, HER2 ECD, HER3 ECD, and HER4 ECD were obtained from Sino Biological Inc. (Beijing, China). They were labelled with a Monolith NT Protein Labelling Kit RED-NHS (Nanotemper, Munich, Germany) and diluted to 250 nM with PBS. Label-free DPBA was diluted at half-concentrations with PBS (100,000–12.21 nM). Label-free EGF was diluted at half-concentrations with PBS (8000–2 nM). Protein samples were mixed with DPBA or EGF and incubated at RT for 5 min. In a competitive assay, EGFR was incubated with DPBA (100 μM) for 60 min at RT before EGF treatment (8000–2 nM). The mixtures were centrifuged at 16,000 × *g* for 5 min and loaded into capillaries. Microscale thermophoresis measurements were performed in a Monolith NT.115 (Nanotemper, Munich, Germany).

### Synthesis of DPBA-1 probe

Synthesis of DPBA-1 was shown in Supplementary Fig. 3d. Briefly, HOBT (15 mg, 0.11 mmol), EDCI (22 mg, 0.11 mmol), and two drops of TEA were added to a solution of S1 (NP) (24.4 mg, 0.1 mmol). The mixture was stirred for 30 min at RT followed by the addition of DPBA (62.2 mg, 0.1 mmol). The reaction was then stirred at RT overnight in dark. Subsequently, the reaction was quenched by the addition of water and extracted with ethyl acetate. The organic layers were combined, washed with brine and dried over anhydrous Na_2_SO_4_. Upon solvent evaporation in vacuo, the residue was purified by flash column (DCM:MeOH = 30:1) to give DPBA-1 as a white solid (26 mg, 30% yield). ^1^H NMR (400 MHz, CDCl_3_) δ 8.22–8.01 (m, 2H), 7.21–7.04 (m, 2H), 4.78 (dd, *J* = 5.2, 2.7 Hz, 2H), 4.70 (d, *J* = 5.6 Hz, 1H), 4.59 (dd, *J* = 15.2, 4.2 Hz, 2H), 4.24 (dd, *J* = 47.7, 7.0 Hz, 1H), 3.63 (dd, *J* = 9.6, 6.2 Hz, 1H), 3.31 (s, 1H), 3.17–2.51 (m, 9H), 2.29–2.09 (m, 2H), 1.97 (m, 5H), 1.74–1.61 (m, 5H), 1.54–1.18 (m, 14H), 0.99–0.79 (m, 10H). ^13^C NMR (101 MHz, CDCl_3_) δ ^13^C NMR (101 MHz, CDCl_3_) δ 173.70, 159.06, 158.95, 157.92, 151.12, 130.46, 127.59, 121.88, 115.91, 109.27, 73.50, 63.67, 57.27, 56.17, 55.12, 54.69, 53.10, 52.60, 51.59, 50.95, 49.27, 48.94, 47.40, 46.30, 45.60, 42.74, 41.87, 40.78, 38.36, 37.44, 36.89, 35.89, 34.82, 33.11, 32.47, 31.72, 30.38, 29.78, 28.30, 26.92, 25.89, 23.71, 22.91, 21.52, 20.21, 19.64, 18.71, 17.43, 16.83, 16.58, 16.13, 16.09, 14.75, 14.57, 13.73, 12.95 and 11.85. LC-MS (electrospray ionization) calculated for C_51_H_73_N_6_O_5_ [M + H] ^+^: 849.5, found 849.5.

### EGFR pull-down assay

The EGFR pull-down assay was performed with DPBA-1, an affinity-based probes.^62^ For total cell pull-down, the cells were treated with DPBA-1, subjected to UV irradiation (302 nm) for 10 min, and lysed with RIPA buffer containing a protease inhibitor cocktail. Then, 200 μg total protein (1 mg/ml) was used in a click chemistry reaction with biotin-N_3_ (50 μM), TBTA (100 μM), TCEP (1 mM), and CuSO_4_ (1 mM), and incubated for 2 h at RT. For the cell lysate pull-down, the cells were lysed by alternate freeze-thaw in liquid nitrogen. Then, 200 μg total protein (1 mg/ml) was incubated with DPBA-1 in the presence or absence of 10× DPBA for 60 min and subjected to UV irradiation (302 nm), and a click chemistry reaction. The reaction was stopped by adding pre-chilled acetone (−20 °C) to precipitate the proteins. After centrifugation at 12,000 r.p.m. and 4 °C for 10 min, the precipitates were dissolved in PBS containing 1% (v/v) SDS and incubated with streptavidin beads for 2 h at RT. The pull-down proteins were eluted with 2× loading buffer at 100 °C for 5 min. The EGFR level was detected by Western blot.

### EGFR dimerization assay

EGFR dimerization was detected by cross-linking assay.^63^ Briefly, cells treated with EGF or DPBA at 4 °C (inhibition of EGFR endocytosis) were incubated with 0.5 mM BS3 at 4 °C for 60 min. The reaction was quenched by incubating with 100 mM glycine at 4 °C for 15 min. Then, cells were lysed in RIPA lysis buffer with protease inhibitor cocktail and EGFR monomer and dimer were separated by 5% SDS-PAGE and detected by Western blot.

### Tumour xenografts in nude mice

The in vivo experiments were approved by the Laboratory Animal Ethics Committee of Jinan University (Guangzhou, China) (No. 201949-02). Primary surgical lung cancer specimens were obtained from the First Affiliated Hospital of Jinan University. Patient data are listed in Supplementary Table 3. Fresh lung cancer tumours were cut into 3–5 mm^3^ sections and subcutaneously inoculated into the axillae of 8-week-old male NOD/SCID mice procured from GemPharmatech Co. Ltd. (Jiangsu, China). The A549, H1650, and H1975 tumour xenograft models were established by suspending 10^7^ cells from each cell line in Matrigel plus PBS at a 2:1 volumetric ratio and subcutaneously injecting the suspensions into the axillae of 6-week-old BALB/c nude mice acquired from Vital River Laboratory Animal Technology (Beijing, China). The mice were randomly divided into the vehicle and DPBA treatment groups when the tumour volumes reached ~100 mm^3^. Vehicle (3% (v/v) DMSO in deionised water) or 25 mg/kg DPBA dissolved in deionised water containing 3% (v/v) DMSO was administered intragastrically once daily for ~1 month. Tumour volumes and body weights were measured every other day. Tumour volumes were calculated as (*a* × *b*^2^)/2, where *a* and *b* are the longest and shortest tumour diameters, respectively. At the end of the experiments, the mice were anesthetised by intraperitoneal injection of 5 ml/kg of 1% (v/v) pentobarbital sodium salt. Their tumours were excised and their blood was drawn for the subsequent measurements.

### Histology and immunohistochemistry (IHC)

For the H&E staining, the tumour tissues were fixed in 4% (v/v) PFA, embedded in paraffin, sliced into 5-µm sections, and stained with H&E. For the IHC assay, the tumour sections were incubated with anti-Ki67 and anti-EGFR antibodies overnight at 4 °C, followed by horse radish peroxidase-conjugated secondary antibodies. The sections were visualized with a DAB Kit and the images were observed under an Olympus BX 53 microscope (Olympus Corp., Tokyo, Japan). All images were statistically analysed in ImagePro Plus v. 6.0 (Media Cybernetics Inc., Rockville, MD, USA).

### Statistical analysis

Each experiment was performed at least three times, and the data were shown as the mean ± standard deviation. Significant differences between two groups were determined using the two-tailed unpaired *t* test, and one-way analysis of variance, followed by Tukey’s post hoc test was used to evaluated significant differences between more than two groups. Differences were considered significant when *P* < 0.05. All statistical data were calculated using the GraphPad Prism 6.0 software.

## Supplementary information

Supplementary Figures

Supplementary Materials

Supplementary information

## Data Availability

The information of 714 compounds is enclosed in Supplementary Table 1. The original datasets are also available from the corresponding author upon request.

## References

[1] Molina JR (2008). Non-small cell lung cancer: epidemiology, risk factors, treatment, and survivorship. Mayo Clin. Proc..

[2] Pao W, Chmielecki J (2010). Rational, biologically based treatment of EGFR-mutant non-small-cell lung cancer. Nat. Rev. Cancer.

[3] Mok TS (2009). Gefitinib or carboplatin–paclitaxel in pulmonary adenocarcinoma. N. Engl. J. Med..

[4] Yu HA, Pao W (2013). Targeted therapies: Afatinib—new therapy option for EGFR-mutant lung cancer. Nat. Rev. Clin. Oncol..

[5] Cross DA (2014). AZD9291, an irreversible EGFR TKI, overcomes T790M-mediated resistance to EGFR inhibitors in lung cancer. Cancer Discov..

[6] Thress KS (2015). Acquired EGFR C797S mutation mediates resistance to AZD9291 in non-small cell lung cancer harboring EGFR T790M. Nat. Med..

[7] Graves LM, Duncan JS, Whittle MC, Johnson GL (2013). The dynamic nature of the kinome. Biochem. J..

[8] Gelsomino F (2013). Epidermal growth factor receptor tyrosine kinase inhibitor treatment in patients with EGFR wild-type non-small-cell lung cancer: the never-ending story. J. Clin. Oncol..

[9] Itchins M, Clarke S, Pavlakis N (2018). Do EGFR tyrosine kinase inhibitors (TKIs) still have a role in EGFR wild-type pre-treated advanced non-small cell lung cancer (NSCLC)?—the shifting paradigm of therapeutics. Transl. Lung Cancer Res..

[10] Weihua Z (2008). Survival of cancer cells is maintained by EGFR independent of its kinase activity. Cancer Cell.

[11] Che TF (2015). Mitochondrial translocation of EGFR regulates mitochondria dynamics and promotes metastasis in NSCLC. Oncotarget.

[12] Cao X, Zhu H, Ali-Osman F, Lo HW (2011). EGFR and EGFRvIII undergo stress- and EGFR kinase inhibitor-induced mitochondrial translocalization: a potential mechanism of EGFR-driven antagonism of apoptosis. Mol. Cancer.

[13] Dykxhoorn DM, Palliser D, Lieberman J (2006). The silent treatment: siRNAs as small molecule drugs. Gene Ther..

[14] Tam YY, Chen S, Cullis PR (2013). Advances in lipid nanoparticles for siRNA delivery. Pharmaceutics.

[15] Sun X (2019). PROTACs: great opportunities for academia and industry. Signal Transduct. Target. Ther..

[16] Cheng M (2020). Discovery of potent and selective epidermal growth factor receptor (EGFR) bifunctional small-molecule degraders. J. Med. Chem..

[17] Gu S (2018). PROTACs: an emerging targeting technique for protein degradation in drug discovery. BioEssays.

[18] Leung EL (2016). Targeting tyrosine kinase inhibitor-resistant non-small cell lung cancer by inducing epidermal growth factor receptor degradation via methionine 790 oxidation. Antioxid. Redox Signal..

[19] Lee JY (2011). Curcumin induces EGFR degradation in lung adenocarcinoma and modulates p38 activation in intestine: the versatile adjuvant for gefitinib therapy. PLoS ONE.

[20] Na YS (2012). YM155 induces EGFR suppression in pancreatic cancer cells. PLoS ONE.

[21] Mao J (2018). Arsenic circumvents the gefitinib resistance by binding to P62 and mediating autophagic degradation of EGFR in non-small cell lung cancer. Cell Death Dis..

[22] Xu SW (2016). Autophagic degradation of epidermal growth factor receptor in gefitinib-resistant lung cancer by celastrol. Int. J. Oncol..

[23] Menard L, Floc’h N, Martin MJ, Cross DAE (2018). Reactivation of mutant-EGFR degradation through clathrin inhibition overcomes resistance to EGFR tyrosine kinase inhibitors. Cancer Res..

[24] Lan P (2011). Synthesis and antiproliferative evaluation of 23-hydroxybetulinic acid derivatives. Eur. J. Med. Chem..

[25] Tan X, Lambert PF, Rapraeger AC, Anderson RA (2016). Stress-induced EGFR trafficking: mechanisms, functions, and therapeutic implications. Trends Cell Biol..

[26] Tomas A, Futter CE, Eden ER (2014). EGF receptor trafficking: consequences for signaling and cancer. Trends Cell Biol..

[27] Heukers R (2013). Endocytosis of EGFR requires its kinase activity and N-terminal transmembrane dimerization motif. J. Cell Sci..

[28] Tong J (2009). Epidermal growth factor receptor phosphorylation sites Ser991 and Tyr998 are implicated in the regulation of receptor endocytosis and phosphorylations at Ser1039 and Thr1041. Mol. Cell Proteom..

[29] Tanaka T (2018). Ligand-activated epidermal growth factor receptor (EGFR) signaling governs endocytic trafficking of unliganded receptor monomers by non-canonical phosphorylation. J. Biol. Chem..

[30] Sigismund S (2005). Clathrin-independent endocytosis of ubiquitinated cargos. Proc. Natl. Acad. Sci. USA.

[31] El-Sayed A, Harashima H (2013). Endocytosis of gene delivery vectors: from clathrin-dependent to lipid raft-mediated endocytosis. Mol. Ther..

[32] Otto GP, Nichols BJ (2011). The roles of flotillin microdomains—endocytosis and beyond. J. Cell Sci..

[33] Burslem GM (2018). The advantages of targeted protein degradation over inhibition: an RTK case study. Cell Chem. Biol..

[34] Sakanyan V (2014). Screening and discovery of nitro-benzoxadiazole compounds activating epidermal growth factor receptor (EGFR) in cancer cells. Sci. Rep..

[35] Iradyan M (2019). Targeting degradation of EGFR through the allosteric site leads to cancer cell detachment-promoted death. Cancers.

[36] Sharma SV, Bell DW, Settleman J, Haber DA (2007). Epidermal growth factor receptor mutations in lung cancer. Nat. Rev. Cancer.

[37] Huang KY (2016). Small molecule T315 promotes casitas B-lineage lymphoma-dependent degradation of epidermal growth factor receptor via Y1045 autophosphorylation. Am. J. Respir. Crit. Care Med..

[38] Alwan HA, van Zoelen EJ, van Leeuwen JE (2003). Ligand-induced lysosomal epidermal growth factor receptor (EGFR) degradation is preceded by proteasome-dependent EGFR de-ubiquitination. J. Biol. Chem..

[39] Sigismund S, Avanzato D, Lanzetti L (2018). Emerging functions of the EGFR in cancer. Mol. Oncol..

[40] Thomas R, Weihua Z (2019). Rethink of EGFR in cancer with its kinase independent function on board. Front. Oncol..

[41] Ahsan A (2012). Wild-type EGFR is stabilized by direct interaction with HSP90 in cancer cells and tumors. Neoplasia (New York, N.Y.).

[42] Uemura T, Kametaka S, Waguri S (2018). GGA2 interacts with EGFR cytoplasmic domain to stabilize the receptor expression and promote cell growth. Sci. Rep..

[43] Zhao XC (2020). Systematic identification of CDC34 that functions to stabilize EGFR and promote lung carcinogenesis. EBioMedicine.

[44] Kitai Y (2017). STAP-2 protein promotes prostate cancer growth by enhancing epidermal growth factor receptor stabilization. J. Biol. Chem..

[45] Ahsan A (2013). Destabilization of the epidermal growth factor receptor (EGFR) by a peptide that inhibits EGFR binding to heat shock protein 90 and receptor dimerization. J. Biol. Chem..

[46] Sigismund S (2008). Clathrin-mediated internalization is essential for sustained EGFR signaling but dispensable for degradation. Dev. Cell.

[47] Tan X, Thapa N, Sun Y, Anderson RA (2015). A kinase-independent role for EGF receptor in autophagy initiation. Cell.

[48] Zwang Y, Yarden Y (2006). p38 MAP kinase mediates stress-induced internalization of EGFR: implications for cancer chemotherapy. EMBO J..

[49] Tomas A (2015). WASH and Tsg101/ALIX-dependent diversion of stress-internalized EGFR from the canonical endocytic pathway. Nat. Commun..

[50] Liao HJ, Carpenter G (2009). Cetuximab/C225-induced intracellular trafficking of epidermal growth factor receptor. Cancer Res..

[51] Brand TM, Iida M, Wheeler DL (2011). Molecular mechanisms of resistance to the EGFR monoclonal antibody cetuximab. Cancer Biol. Ther..

[52] Jaramillo ML (2006). Effect of the anti-receptor ligand-blocking 225 monoclonal antibody on EGF receptor endocytosis and sorting. Exp. Cell Res..

[53] Oksvold MP, Pedersen NM, Forfang L, Smeland EB (2012). Effect of cycloheximide on epidermal growth factor receptor trafficking and signaling. FEBS Lett..

[54] Tanaka T (2018). Ligand-activated epidermal growth factor receptor (EGFR) signaling governs endocytic trafficking of unliganded receptor monomers by non-canonical phosphorylation. J. Biol. Chem..

[55] Yao N (2016). A piperazidine derivative of 23-hydroxy betulinic acid induces a mitochondria-derived ROS burst to trigger apoptotic cell death in hepatocellular carcinoma cells. J. Exp. Clin. Cancer Res..

[56] Yao N (2019). Inhibition of PINK1/Parkin-dependent mitophagy sensitizes multidrug-resistant cancer cells to B5G1, a new betulinic acid analog. Cell Death Dis..

[57] Zhang DM (2012). BBA, a derivative of 23-hydroxybetulinic acid, potently reverses ABCB1-mediated drug resistance in vitro and in vivo. Mol. Pharm..

[58] Ji ZN, Ye WC, Liu GG, Hsiao WL (2002). 23-Hydroxybetulinic acid-mediated apoptosis is accompanied by decreases in bcl-2 expression and telomerase activity in HL-60 Cells. Life Sci..

[59] Salazar G, Gonzalez A (2002). Novel mechanism for regulation of epidermal growth factor receptor endocytosis revealed by protein kinase A inhibition. Mol. Biol. Cell.

[60] Wang J, Yu RK (2013). Interaction of ganglioside GD3 with an EGF receptor sustains the self-renewal ability of mouse neural stem cells in vitro. Proc. Natl. Acad. Sci. USA.

[61] Foerster S (2013). Characterization of the EGFR interactome reveals associated protein complex networks and intracellular receptor dynamics. Proteomics.

[62] Pan S (2016). Target identification of natural products and bioactive compounds using affinity-based probes. Nat. Prod. Rep..

[63] Odintsova E, Voortman J, Gilbert E, Berditchevski F (2003). Tetraspanin CD82 regulates compartmentalisation and ligand-induced dimerization of EGFR. J. Cell Sci..

