# Supplementary material for: Discovery of a novel EGFR ligand DPBA that degrades EGFR and suppresses EGFR-positive NSCLC growth
Source: Signal Transduct Target Ther. 2020 Oct 9;5:214. doi: 10.1038/s41392-020-00251-2 (PMC7544691; doi:10.1038/s41392-020-00251-2)
Supplement: Supplementary file 1 — Supplementary Figures [file 41392_2020_251_MOESM1_ESM.docx]

Supplementary Figures

Fig. S1


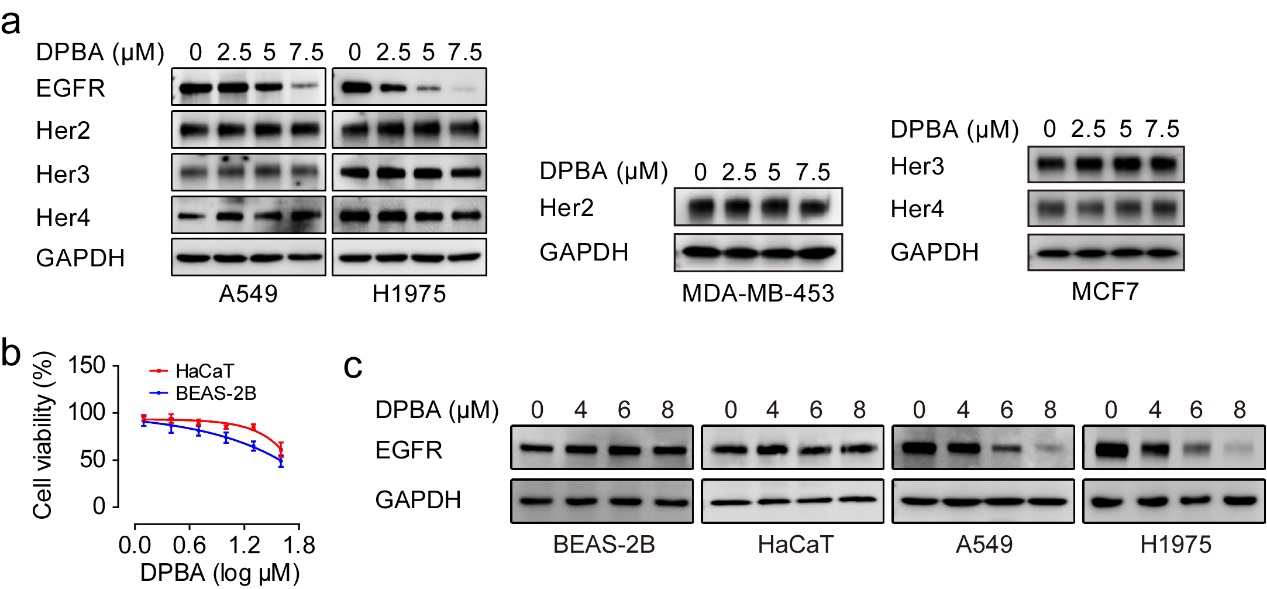


Fig. S2


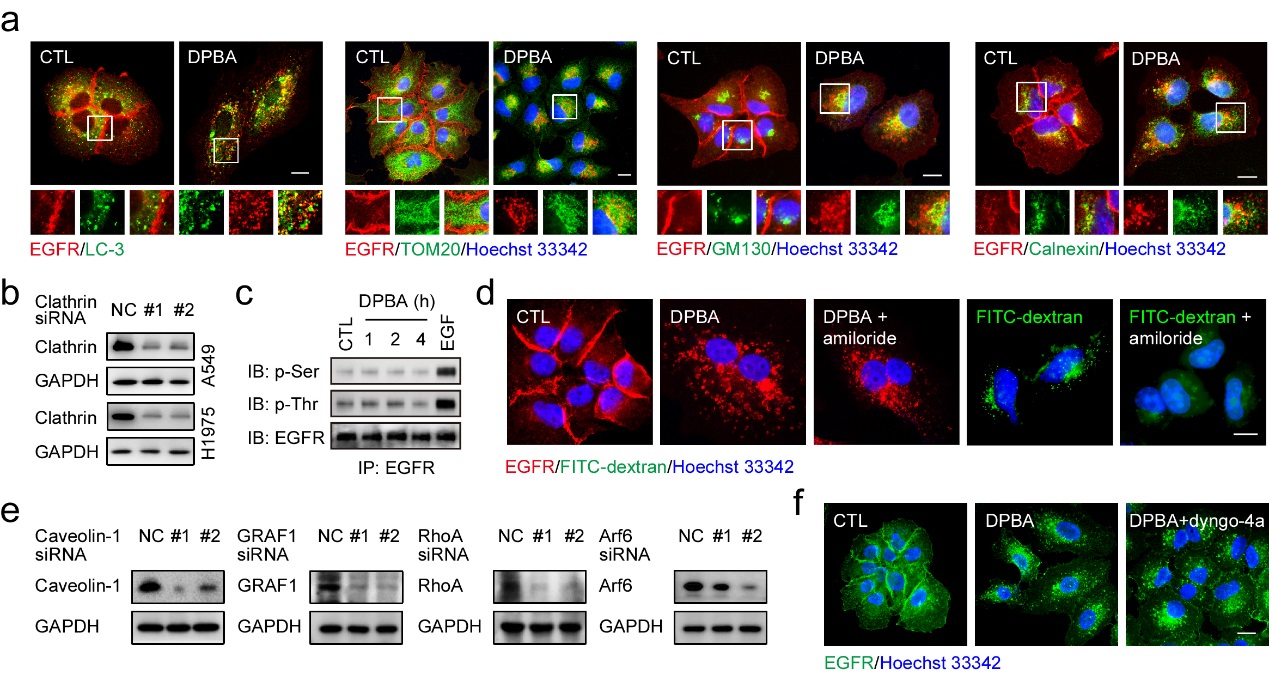


Fig. S3


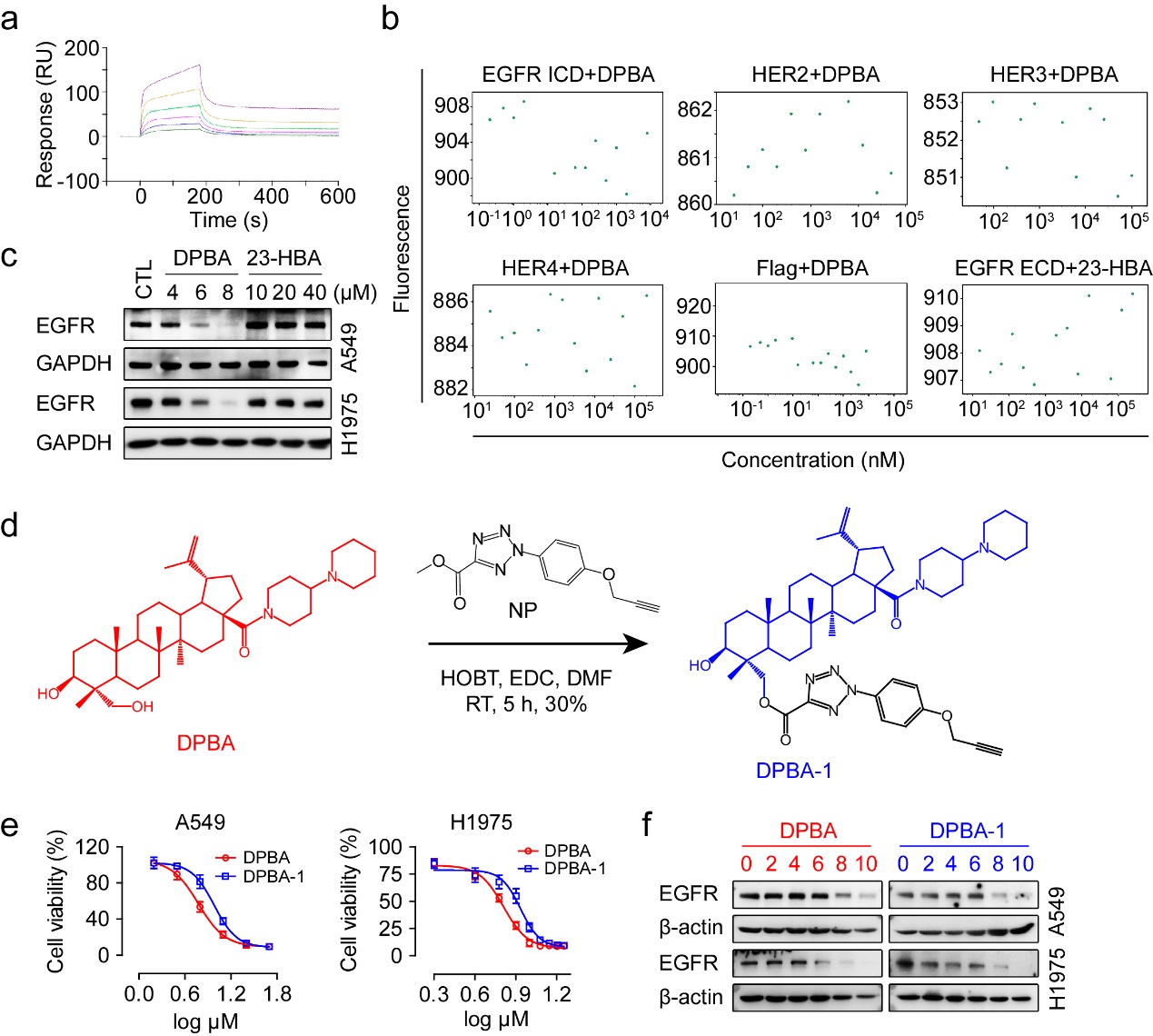


Fig. S4


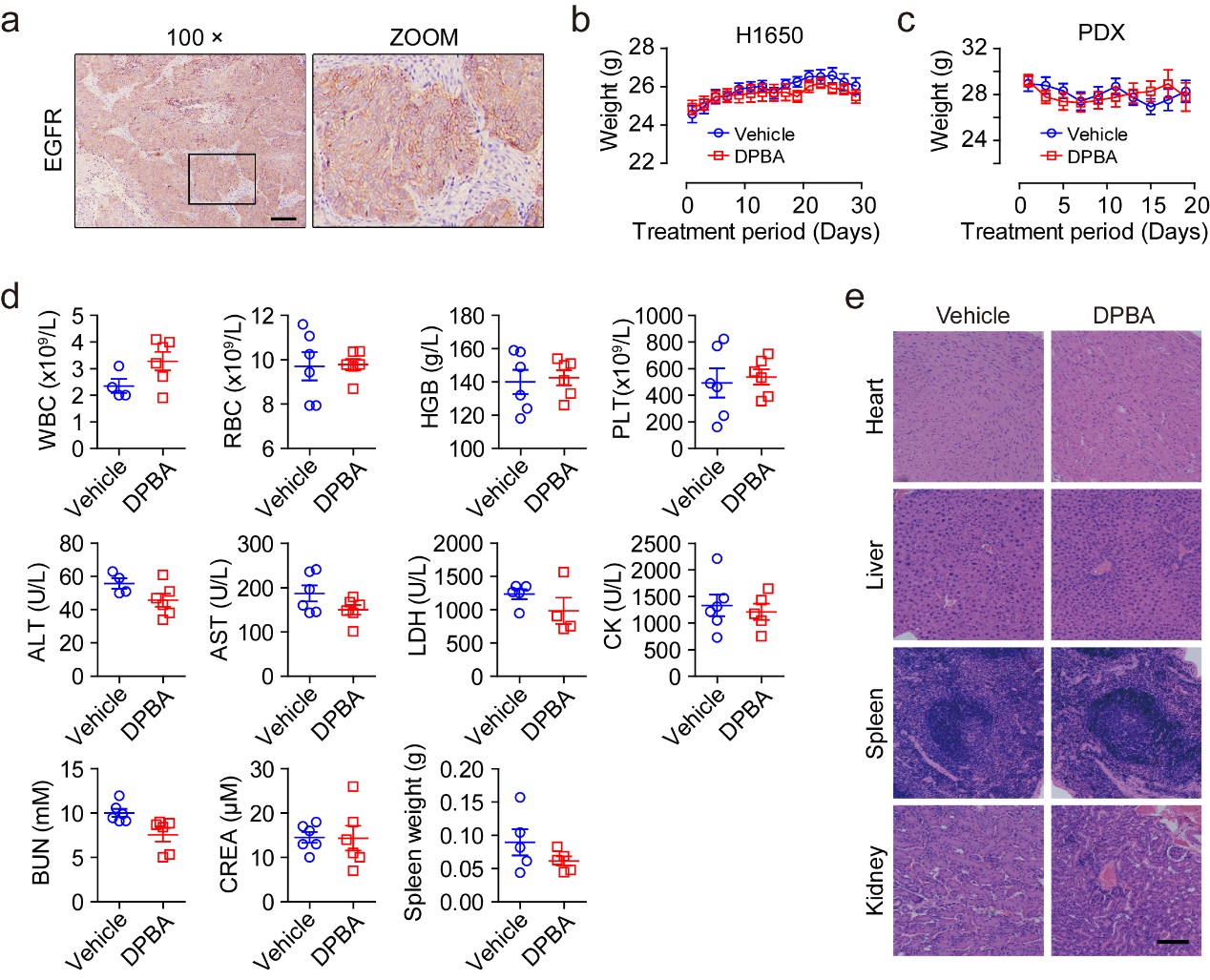


Supplementary Figure legends

**Fig. S1 a** DPBA specifically reduced EGFR protein level. A549, H1975, MDA-MB-453 (Her2-positive), and MCF7 (Her3- and Her4-positive) were treated with DPBA for 24 h. EGFR, Her2, Her3, and Her4 expression levels were detected by Western blot. **b** HaCaT and BEAS-2B were treated with indicated concentrations of DPBA for 24 h. Cell viability was measured by MTT assay, *n* = 3. **c** DPBA did not reduce EGFR protein level in normal cell lines. BEAS-2B, HaCaT, A549, and H1975 were exposed to DPBA (4 μM, 6 μM, or 8 μM) for 24 h. EGFR protein levels were measured by Western blot.

**Fig. S2 a** A549 cells were treated with DPBA (6 μM) for 6 h, colocalization of EGFR and LC-3, TOM20, GM130 or calnexin was detected by immunofluorescence assay (magnification, 630×; scale bar, 10 μm). **b** A549 and H1975 cells were transfected with clathrin siRNA (100 nM) for 48 h, clathrin protein level was detected by Western blot. **c** A549 cells were treated with DPBA (6 μM) for 1, 2, and 4 h or EGF (50 ng/ml) for 5 min. EGFR serine and threonine phosphorylation were detected by EGFR pull-down assay. **d** A549 cells were treated with DPBA (6 μM) in the presence or absence of amiloride (100 μM) for 6 h or FITC-dextran (2 μM) with or without amiloride (100 μM) for 1 h, sub-localization of EGFR or FITC-dextran was detected by immunofluorescence assay (magnification, 630×; scale bar, 10 μm). **e** A549 was transfected with siRNA (100 nM) against caveolin-1, GARF1, RhoA, or Arf6 for 48 h. Proteins levels were measured by Western blot. **f** A549 cells were treated with DPBA in the presence or absence of dyngo-4a (20 μM) for 6 h, sub-localization of EGFR was detected by immunofluorescence assay (magnification: 630×; scale bar: 10 μm).

**Fig. S3 a** The interaction between EGF and EGFR ECD was measured by BIACORE. **b** The interaction between DPBA and EGFR ICD, HER2 ECD, HER3 ECD, HER4 ECD or flag tag in EGFR ECD, as well as interaction between EGFR ECD and 23-HBA were measured by MST. **c** A549 and H1975 cells were treated with DPBA or 23-HBA for 24 h, EGFR protein level was detected by Western blot. **d** Synthesis of DPBA-1 probe. **e** A549 and H1975 cells were treated with indicated concentrations of DPBA or DPBA-1 for 24 h, cell viability was measured by MTT assay, *n* = 3. **f** A549 and H1975 cells were treated with indicated concentrations of DPBA or DPBA-1 for 12 h, EGFR protein level was detected by Western blot.

**Fig. S4 a** Immunohistochemistry staining for EGFR of primary lung cancer PDX (magnification, 100×; scale bar, 200 μm). **b** Body weight curve of mice bearing H1650 xenografts. **c** Body weight curve of mice bearing primary NSCLC PDX. **d** Examination of routine blood indices, serum biochemical indices, and spleen weight of nude mice treated with DPBA (25 mg/kg). **e** H&E staining of the main organs of nude mice (magnification, 200×; scale bar, 400 μm).
