# Supplementary material for: Discovery of a novel EGFR ligand DPBA that degrades EGFR and suppresses EGFR-positive NSCLC growth
Source: Signal Transduct Target Ther. 2020 Oct 9;5:214. doi: 10.1038/s41392-020-00251-2 (PMC7544691; doi:10.1038/s41392-020-00251-2)
Supplement: Supplementary file 2 — Supplementary Materials [file 41392_2020_251_MOESM2_ESM.docx]

Supplementary Materials for

Discovery of a novel EGFR ligand DPBA that degrades EGFR and suppresses EGFR-positive NSCLC growth

Nan Yao^1, 2, a^, Chen-Ran Wang^1, 2, a^, Ming-Qun Liu^1^, Ying-Jie Li^1^, Wei-Min Chen^1^, Zheng-Qiu Li^1^, Qi Qi^3^, Jin-Jian Lu^4^, Chun-Lin Fan^1, 2^, Min-Feng Chen^1, 2^, Ming Qi^1, 2^, Xiao-Bo Li^1, 2^, Jian Hong^3^, Dong-Mei Zhang^1, 2, *^, Wen-Cai Ye^1, 2, *^

^1^ College of Pharmacy, Jinan University, Guangzhou, China

^2^ Guangdong Province Key Laboratory of Pharmacodynamic Constituents of Traditional Chinese Medicine and New Drugs Research, Jinan University, Guangzhou, China

^3^ School of Medicine, Jinan University, Guangzhou, China

^4^ State Key Laboratory of Quality Research in Chinese Medicine, Institute of Chinese Medical Sciences, University of Macau, Macao, China

^a^ These authors contributed equally to this work.

***Corresponding Authors:**

Prof. Wencai Ye, College of Pharmacy, Jinan University, 601 Huangpu Avenue. West, Guangzhou 510632, China; Phone: +86-20-85220936; E-mail: chywc@aliyun.com.

Prof. Dongmei Zhang, College of Pharmacy, Jinan University, 601 Huangpu Avenue. West, Guangzhou 510632, China; Phone: +86-20-85222653; E-mail: dmzhang701@jnu.edu.cn.

**This PDF file includes:**

Figures S1 to S4

Tables S2 to S3

**Other Supplementary Materials for this manuscript include the following:**

Table S1

Figure. S1.


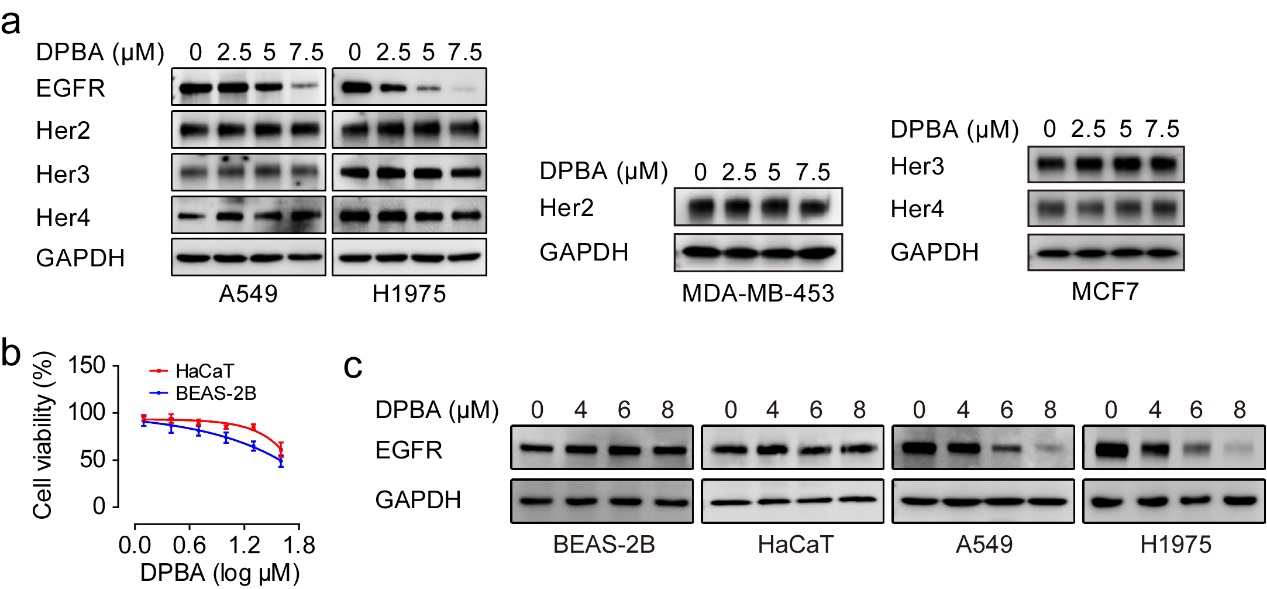


**Figure S1 a** DPBA specifically reduced EGFR protein level. A549, H1975, MDA-MB-453 (Her2-positive), and MCF7 (Her3- and Her4-positive) were treated with DPBA for 24 h. EGFR, Her2, Her3, and Her4 expression levels were detected by Western blot. **b** HaCaT and BEAS-2B were treated with indicated concentrations of DPBA for 24 h. Cell viability was measured by MTT assay, *n* = 3. **c** DPBA did not reduce EGFR protein level in normal cell lines. BEAS-2B, HaCaT, A549, and H1975 were exposed to DPBA (4 μM, 6 μM, or 8 μM) for 24 h. EGFR protein levels were measured by Western blot.

Figure. S2.


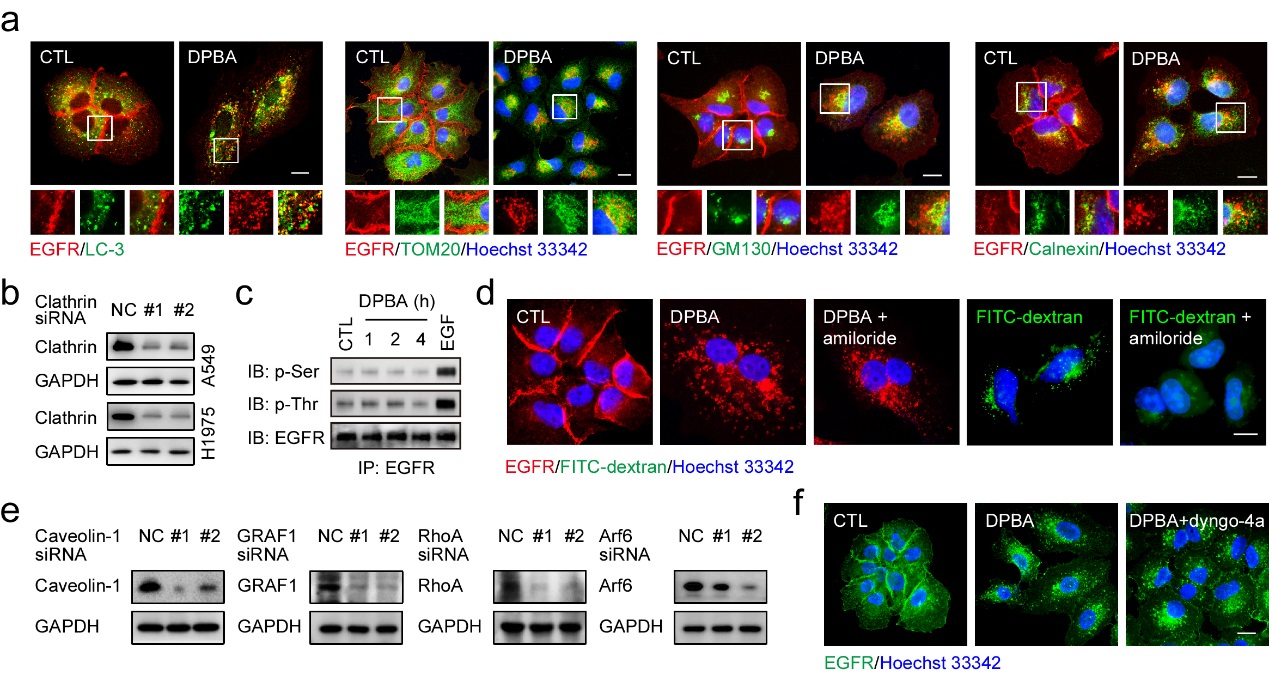


**Figure S2 a** A549 cells were treated with DPBA (6 μM) for 6 h, colocalization of EGFR and LC-3, TOM20, GM130 or calnexin was detected by immunofluorescence assay (magnification, 630×; scale bar, 10 μm). **b** A549 and H1975 cells were transfected with clathrin siRNA (100 nM) for 48 h, clathrin protein level was detected by Western blot. **c** A549 cells were treated with DPBA (6 μM) for 1, 2, and 4 h or EGF (50 ng/ml) for 5 min. EGFR serine and threonine phosphorylation were detected by EGFR pull-down assay. **d** A549 cells were treated with DPBA (6 μM) in the presence or absence of amiloride (100 μM) for 6 h or FITC-dextran (2 μM) with or without amiloride (100 μM) for 1 h, sub-localization of EGFR or FITC-dextran was detected by immunofluorescence assay (magnification, 630×; scale bar, 10 μm). **e** A549 was transfected with siRNA (100 nM) against caveolin-1, GARF1, RhoA, or Arf6 for 48 h. Proteins levels were measured by Western blot. **f** A549 cells were treated with DPBA in the presence or absence of dyngo-4a (20 μM) for 6 h, sub-localization of EGFR was detected by immunofluorescence assay (magnification: 630×; scale bar: 10 μm).

Figure. S3.


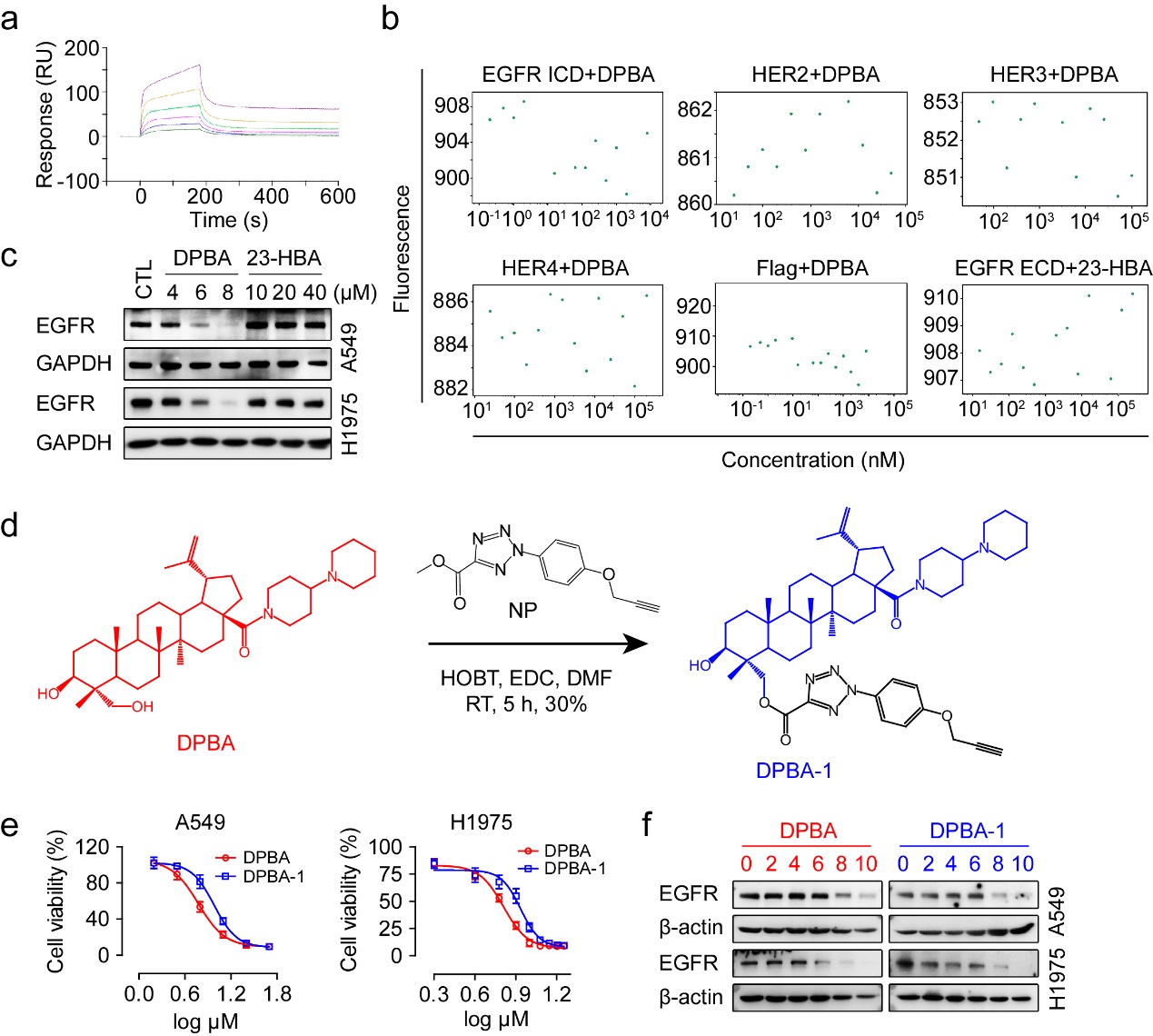


**Figure S3 a** The interaction between EGF (200, 100, 50, 25, 12.5, and 6.25 μM) and EGFR ECD was measured by BIACORE. **b** The interaction between DPBA and EGFR ICD, HER2 ECD, HER3 ECD, HER4 ECD or flag tag in EGFR ECD, as well as interaction between EGFR ECD and 23-HBA were measured by MST. **c** A549 and H1975 cells were treated with DPBA or 23-HBA for 24 h, EGFR protein level was detected by Western blot. **d** Synthesis of DPBA-1 probe. **e** A549 and H1975 cells were treated with indicated concentrations of DPBA or DPBA-1 for 24 h, cell viability was measured by MTT assay, *n* = 3. **f** A549 and H1975 cells were treated with indicated concentrations of DPBA or DPBA-1 for 12 h, EGFR protein level was detected by Western blot.

Figure. S4.


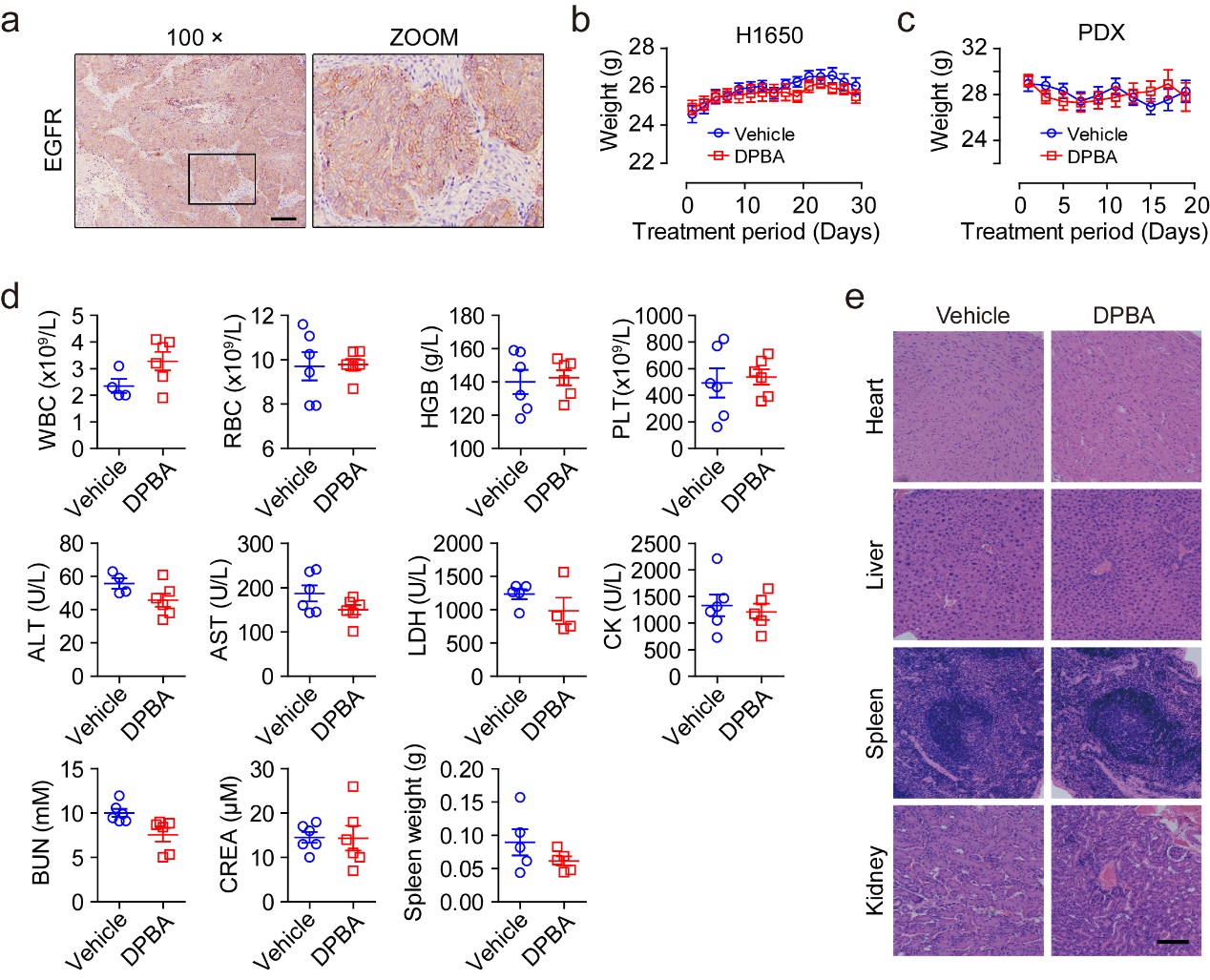


**Figure S4 a** Immunohistochemistry staining for EGFR of primary lung cancer PDX (magnification, 100×; scale bar, 200 μm). **b** Body weight curve of mice bearing H1650 xenografts. **c** Body weight curve of mice bearing primary NSCLC PDX. **d** Examination of routine blood indices, serum biochemical indices, and spleen weight of nude mice treated with DPBA (25 mg/kg). **e** H&E staining of the main organs of nude mice (magnification, 200×; scale bar, 400 μm).

Table S2.

**Table S2:** siRNA sequences of targeted proteins

| **Name** | **No.** | **Sequences** |
| --- | --- | --- |
| Dynamin-2 | #1 | CCGAATCAATCGCATCTTC |
|  | #2 | GACATGATCCTGCAGTTCA |
| Clathrin | #1 | CCTGCGGTCTGGAGTCAAC |
|  | #2 | GAAGAACTCTTTGCCCGGAAATTTA |
| Caveolin-1 | #1 | GGCCAGCUUCACCACCUUC |
|  | #2 | AGACGAGCUGAGCGAGAAGCA |
| Flotillin-1 | #1 | GCAGAGAAGUCCCAACUAA |
|  | #2 | GUGGUUAGCUACACUCUGA |
| Arf6 | #1 | GCACCGCAUUAUCA AUGACCGUU |
|  | #2 | CGGUCAUUGAUAAUGCGGUGCUU |
| GRAF1 | #1 | GUA AUCUGUGCUGAAUGGGAGAUAA |
|  | #2 | CCACUCAUGAUGUACCAGUUUCAAA |
| RhoA | #1 | AUGGAAAGCAGGUAGAGUU |
|  | #2 | TACCCAGATACCGATGTTATA |

Table S3.

**Table S3:** Clinical characteristics of the primary lung cancer

| **Characteristics** | **Lung cancer patient** |
| --- | --- |
| Gender/Age (yr) | Male/60 |
| Date of diagnosis | 2019/6/25 |
| Tumor type | Squamous cell carcinoma |
| Clinical stage | T3N0M0 |
| EGFR expression | Positive |
| Treatment status | Post-operative chemotherapy |
| Clinical metastasis | None |
